# Global, regional, and national trends in hypertensive heart disease burden due to high BMI: a 30-year analysis using GBD 2021 data with projections to 2035

**DOI:** 10.3389/fpubh.2026.1701954

**Published:** 2026-01-30

**Authors:** Muhammad Babar Khawar, Kaleem Maqsood, Rui Sang, Javeria Malik, Ali Afzal, Azeem Saeed, Farwa Liaqat, Humera Naveed, Akasha Fiaz, Chatchai Muanprasat, Jing Zhou

**Affiliations:** 1 Health Management Center, Affiliated Hospital of Yangzhou University, Yangzhou, Jiangsu, China; 2 School of Basic Medical Sciences & School of Public Health, Faculty of Medicine, Yangzhou University, Yangzhou, China; 3 Department of Biology, Lahore Garrison University, Lahore, Pakistan; 4 Institute of Zoology, University of the Punjab, Lahore, Pakistan; 5 Shenzhen Institute of Advanced Technology, Chinese Academy of Sciences, Shenzhen, China; 6 University of Chinese Academy of Sciences, Beijing, China; 7 Allama Iqbal Medical College/Jinnah Hospital, Lahore, Punjab, Pakistan; 8 Chakri Naruebodindra Medical Institute, Faculty of Medicine Ramathibodi Hospital, Mahidol University, Samut Prakarn, Thailand

**Keywords:** epidemiology, global burden of disease, hypertensive heart disease, mortality, obesity

## Abstract

**Background:**

High body mass index (BMI)-related hypertensive heart disease (HHD) is increasingly prevalent worldwide. Using Global Burden of Disease (GBD) 2021 data, we analyzed the changes in disability-adjusted life years (DALYs) and age-standardized rate (ASR) of mortality (ASMR) due to high BMI from 1990 to 2021.

**Methods:**

HHD data on high BMI were obtained from GBD 2021 at global, regional, and country levels. Age-standardized DALYs (ASDR) and deaths (ASMR) were calculated, with trends analyzed based on gender, age, and region. The autoregressive integrated moving average (ARIMA) model was used to project the burden through 2035, while the estimated annual percentage change (EAPC) was used to assess future trends.

**Results:**

From 1990 to 2021, global DALYs increased from 5.67 million to 12.55 million (a 1.81% rise in ASR), and deaths rose from 240,000 to 594,000. Men showed an 8.28% increase in DALYs, while women’s burden remained stable with a slight ASR decline. The highest burden was observed in those aged 80 and older, with DALYs increasing from 1243.80 to 1604.32. Projections suggest gradual decreases in DALYs and ASMR by 2035, although high BMI-related HHD remains a major public health concern.

**Conclusion:**

High BMI intensifies HHD prevalence, particularly among men and older adults. Despite projected minor decreases by 2035, rising obesity underscores the ongoing need for public health interventions.

## Introduction

Obesity is a controllable metabolic risk factor that develops from a persistent imbalance between energy intake and abnormal fat accumulation (1). Obesity is strongly associated with an increased risk of metabolic and systemic complications, including type 2 diabetes, hypertension, cardiovascular disease, renal impairment, chronic respiratory conditions, hepatic cirrhosis, and multiple malignancies (2–6). From 1990 to 2017, more than 70 countries experienced a twofold increase in the health burden associated with high body mass index (BMI), and it is projected to rise further (7). The global prevalence of hypertension has increased markedly, with the number of affected adults rising from 594 million in 1975 to 1.13 billion in 2015 (8).

Hypertensive heart disease (HHD) is a pathological condition secondary to hypertension, manifested by structural alterations such as left ventricular hypertrophy, functional deficits including systolic and diastolic dysfunction, and other compensatory modifications within the cardiovascular system (9). By 2020, HHD and other non-communicable cardiovascular diseases had emerged as the leading causes of mortality and disability worldwide (10). Non-communicable diseases account for 41 million deaths every year, which are equivalent to 74% of all deaths globally. Cardiovascular disease accounts for approximately 17.9 million deaths per year, nearly one-third of the total deaths (11). In 2021, there were 12.5 million HHD cases globally, resulting in 1.332 million deaths and 25.4622 million DALYs (12).

Obesity contributes to HHD by leptin-mediated renin elevation (13), sympathetic system overactivity, which disrupts the regulation of renin–aldosterone levels, leading to cardiac fibrosis and endothelial dysfunction (13, 14), and by causing inflammation and lipid accumulation in tissues, thereby disrupting several intracellular pathways (15, 16). The age-dependent progression and long-term nature of HHD make it a pressing public health challenge and impose stantial healthcare costs (17, 18). This study is the first to project global HHD burden attributable to high BMI using GBD 2021 data and ARIMA modeling. ARIMA models represent changing trends, periodic variations, and random fluctuations in time series data and are commonly used to model the dependencies over time. They have been used in epidemiology to predict the trends of diseases, such as influenza mortality (19) and other diseases (20, 21). This study used ARIMA to extrapolate future trends in the burden of hypertensive heart diseases attributable to high BMI using GBD data.

To mitigate the substantial social and economic burden associated with high BMI, particularly HHD, it is essential to formulate evidence-based policies and implement effective strategies for prevention and control in a timely manner. This requires an accurate understanding of the current burden and temporal patterns of high BMI-related HHD at the global level. There have been studies that highlighted the global burden of HHD based on the Global Burden of Disease, Injuries, and Risk Study (GBD) 2019 and 2021 (9, 12). However, to date, no comprehensive GBD studies have provided detailed estimates of HHD burden due to high BMI. Our study aims to address this gap by studying the global, regional, and national patterns in HHD prevalence, incidence, deaths, and disability-adjusted life years (DALYs) due to high BMI. To date, no comprehensive studies have provided detailed estimates of this burden. The GBD 2021 study, owing to its updated population-based data, comprehensive global coverage, integration of diverse data sources by methods like the DisMod-MR 2.1 model, and other key methodological refinements (22), offers a systematic and scientific assessment of the epidemiological profile of 87 risk factors and 369 diseases and injuries across 204 countries and territories from 1990 to 2021 (23, 24).

In this study, we provide a comprehensive analysis of the burden of HHD worldwide caused by a high BMI and based on the latest GBD 2021 data. It reports on mortality and DALYs by age group, sex, sociodemographic index, and country or territory in 204 countries and territories over 1990–2021, with projections extending to 2035. This study provides up-to-date, detailed estimates of the global HHD burden attributable to high BMI and identifies temporal and demographic trends, expecting to reveal rising and unequal burdens across regions to guide public health policies and resource allocation for HHD management and close gaps in HHD disparities.

## Methods

### Data source

GBD 2021 is an international study that has consistently assessed the health burden of countries worldwide since 1990. This study also provides major epidemiological indicators, such as incidence, prevalence, and mortality rates, as well as overall health indicators, such as DALYs, which combine years of life lost due to early death and years lived with disability. Estimates are based on prevalence information and disability weights and are computed using life tables. Each year, updates are made to include new diseases, more data sources, and improved methods of analysis. The results are available through GBD Compare,^1^ and data sources used as input are on the Global Health Data Exchange.^2^ The study follows the GATHER principles, ensuring proper and clear health estimates (24).

### Definition of concepts

The definition of HHD that was included in the GBD 2021 study corresponds to that of the initial study. High BMI was considered above 25 kg/cm^2^ by calculating kilograms to the square of the centimeter height (kg/cm^2^) among those above 20 years. The socio-demographic index (SDI) is the geometric mean of 0 to 1 indices of total fertility rate under the age of 25, mean education for those ages 15 and older, and lag distributed income per capita. As a composite, a location with an SDI of 0 would have a theoretical minimum level of development relevant to health, while a location with an SDI of 1 would have a theoretical maximum level.

### Statistical analysis

The estimated annual percentage change (EAPC) was utilized to analyze trends in ASRs during the study period (1990–2021). This metric was derived by applying a linear regression model to the natural logarithm of ASRs over time. The 95% confidence interval (CI) for EAPC was determined using the formula 100 [exp(b)–1]. The interpretation of EAPC trends was based on its 95% CI:

If b > 0 and the lower bound of the CI (LCI) > 0, the disease burden was considered to be increasing. If b < 0 and the upper bound of the CI (UCI) < 0, the disease burden was interpreted as decreasing. If the 95% CI included 0, the disease burden was considered stable over time (25, 26).

Subgroup analyses performed as part of this study were descriptive, and they sought to summarize the patterns and trends in the burden of HHD attributable to high BMI across different genders, countries, and regions. No hypothesis testing or statistical inference was conducted, so no multiple comparison adjustments were made. The ARIMA (AutoRegressive Integrated Moving Average) model was used to forecast the ASDR and ASMR for HHD attributable to high BMI, as it effectively captures temporal autocorrelation and trends in epidemiological time series data, making it suitable for disease burden projections. Stationarity was achieved by differencing the time series data, and this was confirmed via the Augmented Dickey-Fuller test. Model parameters were selected using the Autocorrelation Function (ACF), partial autocorrelation function, and information criteria such as AIC and BIC. Model diagnostics, including the Ljung-Box test, validated the adequacy of residuals, ensuring no autocorrelation remained. Forecasts were generated along with 95% confidence intervals to express uncertainty. Model performance metrics, including MAPE, RMSE, and AIC/BIC, indicated a strong fit and forecasting reliability. All data analysis and visualization were carried out using R statistical software (version 4.5.1) and GraphPad Prism (version 8.0). The analysis used R packages including tidyverse, dplyr, psych, summarytools, broom, ggplot2, forecast, tseries, and sf for data management, statistical modeling, visualization, and forecasting, with results rounded to two decimal places for consistency. Statistical outputs were also rounded to two decimal places to enhance clarity.

## Results

Between 1990 and 2021, global DALYs due to HHD associated with high BMI increased from 5.67 million to 12.55 million, while deaths increased from 240,000 to 594,000. Men had a greater increase in burden compared to women. In addition, the rates were notably highest in those aged 80 years and older. There were regional differences, with a significant increase in high-income and low-middle SDI regions. Predictions until 2035 indicate a slight decline; however, high BMI-linked HHD is expected to remain a major global health problem.

### Global trends

**Figure 1:**
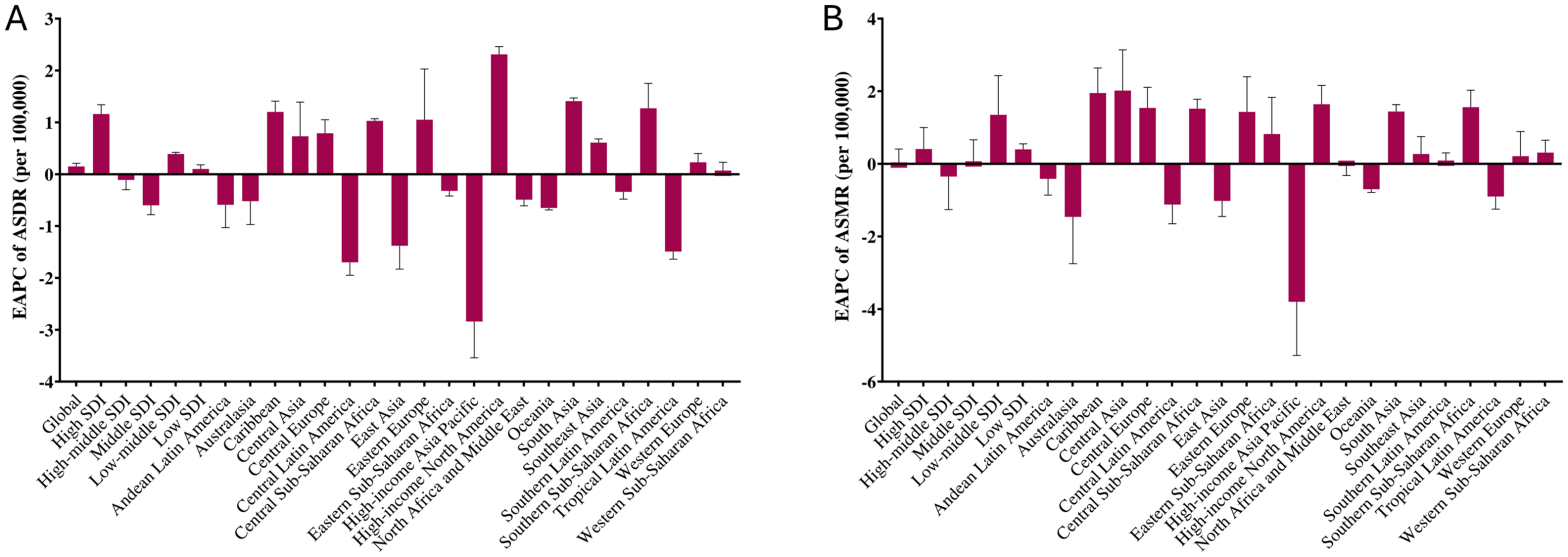
Estimated annual percentage change (EAPC) with 95% confidence intervals, in ASRs of disability-adjusted life years (ASDR) **(A)** and mortality **(B)** attributable to hypertensive heart disease (HHD) due to high body mass index (BMI) across GBD regions from 1990 to 2021. Positive EAPC values indicate increasing trends, whereas negative values indicate decreasing trends over the study period.

The global burden of HHD due to high BMI has shown substantial trends from 1990 to 2021. For DALYs, the ASDR per 100,000 slightly increased from 144.72 in 1990 to 147.33 in 2021, reflecting a 1.81% increase and an EAPC of 0.15% (Table 1; Figure 1A). Similarly, deaths due to HHD caused by high BMI also increased significantly. The ASMR per 100,000 rose from 6.83 in 1990 to 7.21 in 2021, with a 5.62% increase and a modest EAPC of 0.04% (Table 2; Figure 1B).

**Table 1:** Disability-adjusted life years from hypertensive heart disease attributable to high body mass index across global burden of disease regions.

Location	No. x10^3^ 2021 (95% UI)	ASR, per 100,000 in 1990 (95% UI)	ASR, per 100,000 in 2021 (95% UI)	PC, % (1990–2021) (95% UI)	EAPC, % (95% CI) (1990–2021)
Global	12551.75(9489.12,15451.02)	144.72(106.21,182.76)	147.33(109.06,183.45)	1.81(−10.43,20.86)	0.15(0.10,0.21)
Male	5646.14(4247.05,6976.63)	131.37(96.12,166.66)	142.25(104.32,179.41)	8.28(−4.28,24.63)	0.40(0.33,0.47)
Female	6905.61(5009.10,8756.18)	154.37(107.15,198.32)	150.26(109.60,190.15)	−2.66(−17.42,21.00)	−0.04(−0.09,0.01)
SDI regions					
High SDI	1888.25(1342.87,2367.63)	79.52(59.95,98.57)	97.25(76.46,115.81)	22.29(11.51,36.40)	1.16(0.99,1.34)
High-middle SDI	2296.32(1583.47,3023.14)	127.41(92.84,165.58)	118.94(81.23,157.67)	−6.65(−21.63,11.47)	−0.11(−0.30,0.07)
Middle SDI	4255.95(3088.32,5431.40)	197.12(132.36,261.86)	163.33(112.87,214.45)	−17.14(−30.15,6.11)	−0.60(−0.78,-0.42)
Low-middle SDI	2774.97(2163.09,3408.02)	174.50(122.01,231.14)	192.73(147.45,244.13)	10.44(−6.38,34.19)	0.39(0.36,0.42)
Low SDI	1318.23(895.03,1781.08)	233.39(132.99,326.55)	249.65(164.84,328.72)	6.97(−12.14,43.57)	0.10(0.02,0.18)
GBD regions					
Andean Latin America	51.49(36.48,68.30)	125.44(93.20,159.74)	87.42(60.88,117.12)	−30.31(−42.75,-15.93)	−0.59(−1.03,-0.14)
Australasia	13.46(8.33,17.82)	29.57(20.80,38.89)	24.32(16.24,31.00)	−17.77(−26.08,-10.49)	−0.52(−0.97,-0.07)
Caribbean	124.03(94.91,153.88)	180.52(138.74,228.31)	230.58(176.69,285.28)	27.73(10.26,45.58)	1.20(1.00,1.41)
Central Asia	177.45(135.76,223.72)	182.97(142.27,227.00)	225.17(167.21,286.40)	23.06(1.92,48.41)	0.73(0.09,1.39)
Central Europe	576.78(392.43,742.47)	234.60(179.04,292.21)	256.77(182.69,324.76)	9.45(−3.60,20.09)	0.79(0.54,1.05)
Central Latin America	248.38(179.78,324.94)	163.52(120.71,206.61)	101.12(71.30,132.94)	−38.16(−48.28,-27.78)	−1.70(−1.95,-1.44)
Central Sub-Saharan Africa	292.00(171.82,431.34)	390.74(195.43,580.93)	546.75(322.13,825.58)	39.93(4.77,109.17)	1.03(0.98,1.07)
East Asia	2468.56(1553.65,3558.54)	180.32(105.12,256.67)	120.29(72.71,178.97)	−33.29(−50.29,0.74)	−1.38(−1.83,-0.94)
Eastern Europe	312.74(239.27,385.02)	61.10(50.73,70.84)	90.73(71.47,110.51)	48.49(29.08,65.94)	1.05(0.08,2.03)
Eastern Sub-Saharan Africa	521.32(338.09,709.15)	311.70(169.90,439.48)	298.05(180.48,414.03)	−4.38(−21.66,29.63)	−0.32(−0.42,-0.22)
High-income Asia Pacific	93.54(44.90,149.94)	47.35(29.06,66.74)	18.25(11.86,25.80)	−61.46(−65.68,-50.47)	−2.84(−3.54,-2.13)
High-income North America	983.53(778.73,1178.29)	95.99(77.80,113.60)	172.21(142.82,201.13)	79.40(61.70,102.06)	2.31(2.17,2.46)
North Africa and the Middle East	1910.27(1455.25,2382.73)	522.42(363.93,686.68)	440.20(317.57,558.72)	−15.74(−31.63,8.79)	−0.49(−0.61,-0.36)
Oceania	21.67(14.66,31.79)	290.91(188.24,402.99)	243.69(171.23,353.90)	−16.23(−35.32,15.17)	−0.65(−0.69,-0.60)
South Asia	1490.18(1049.06,2077.93)	67.92(38.40,98.92)	101.32(70.52,143.72)	49.19(8.57,131.89)	1.41(1.35,1.47)
Southeast Asia	1159.65(809.40,1490.33)	141.20(86.26,193.98)	168.45(118.20,217.74)	19.30(−1.73,61.77)	0.61(0.55,0.68)
Southern Latin America	115.62(75.59,149.42)	158.65(118.36,198.15)	129.88(86.97,166.68)	−18.13(−30.27,-10.26)	−0.34(−0.48,-0.20)
Southern Sub-Saharan Africa	328.75(265.70,399.28)	412.08(317.08,535.70)	578.12(437.99,722.53)	40.30(23.85,58.78)	1.27(0.80,1.75)
Tropical Latin America	366.92(286.76,442.70)	231.50(183.38,281.97)	143.42(110.18,174.44)	−38.05(−44.26,-31.48)	−1.49(−1.64,-1.35)
Western Europe	667.21(285.61,993.37)	66.72(44.26,88.38)	59.46(31.29,83.16)	−10.88(−29.37,-0.71)	0.23(0.05,0.40)
Western Sub-Saharan Africa	628.19(371.11,822.11)	272.01(178.00,372.99)	296.30(180.82,399.61)	8.93(−27.22,44.17)	0.07(−0.10,0.23)

ASR, age-standardized rate; BMI, body mass index; DALYs, disability-adjusted life years; EAPC, estimated annual percentage change; GBD, Global Burden of Disease; HHD, hypertensive heart disease; PC, percentage change; UI, uncertainty interval.

**Table 2:** Deaths from hypertensive heart disease linked to high body mass index by global region.

Location	No. x10^3^ 2021 (95% UI)	ASR, per 100,000 in 1990 (95% UI)	ASR, per 100,000 in 2021 (95% UI)	PC, % (1990–2021) (95% UI)	EAPC, % (1990–2021) (95% CI)
Global	594.90(362.92,804.91)	6.83(4.37,9.32)	7.21(4.23,9.94)	5.62(−8.50,23.15)	0.04(−0.33,0.41)
Male	243.06(159.71,323.10)	5.97(3.74,8.25)	6.69(3.98,9.30)	12.03(−3.50,28.94)	0.55(0.47,0.63)
Female	351.84(201.12,494.20)	7.37(4.60,10.12)	7.53(4.36,10.53)	2.19(−14.39,22.79)	0.20(0.15,0.25)
SDI regions					
High SDI	101.72(51.59,144.02)	3.88(2.28,5.34)	4.38(2.59,5.87)	12.81(2.70,24.89)	0.41(−0.18,1.00)
High-middle SDI	132.67(67.11,191.44)	6.62(3.92,9.36)	7.00(3.38,10.17)	5.75(−16.60,24.53)	−0.35(−1.26,0.58)
Middle SDI	196.80(122.49,276.06)	9.61(5.53,13.78)	8.30(4.53,12.23)	−13.64(−28.78,9.62)	0.07(−0.52,0.66)
Low-middle SDI	113.74(81.65,147.94)	8.11(5.03,11.49)	8.96(5.90,12.38)	10.41(−7.34,31.72)	1.35(0.28,2.43)
Low SDI	49.04(31.97,64.99)	10.04(5.58,14.81)	11.27(6.94,15.88)	12.25(−9.39,48.96)	0.40(0.25,0.55)
GBD regions					
Andean Latin America	2.48(1.38,3.57)	5.86(3.68,8.28)	4.38(2.37,6.42)	−25.23(−40.36,-9.62)	−0.41(−0.86,0.03)
Australasia	0.84(0.34,1.22)	1.63(0.85,2.38)	1.34(0.62,1.90)	−17.72(−30.00,-8.22)	−1.46(−2.75,-0.16)
Caribbean	5.46(3.58,7.23)	7.81(5.33,10.44)	9.98(6.59,13.15)	27.86(11.36,44.40)	1.95(1.27,2.64)
Central Asia	7.83(5.33,10.30)	8.24(5.49,11.12)	11.36(7.06,15.74)	37.84(9.32,70.34)	2.02(0.92,3.14)
Central Europe	34.42(18.12,47.35)	12.18(7.52,16.66)	14.52(7.94,19.75)	19.24(0.34,31.03)	1.54(0.98,2.11)
Central Latin America	12.77(7.67,17.93)	8.45(5.15,11.79)	5.40(3.14,7.69)	−36.07(−46.58,-25.25)	−1.12(−1.65,-0.59)
Central Sub-Saharan Africa	11.19(6.48,17.05)	17.42(8.38,27.05)	26.46(14.82,40.53)	51.93(11.46,123.55)	1.52(1.26,1.78)
East Asia	129.83(67.19,204.26)	9.53(4.68,14.49)	6.89(3.18,11.52)	−27.70(−49.07,7.40)	−1.02(−1.45,-0.60)
Eastern Europe	15.86(10.10,21.12)	2.50(1.86,3.13)	4.49(2.96,5.91)	79.71(49.82,102.59)	1.43(0.46,2.40)
Eastern Sub-Saharan Africa	19.48(11.56,27.47)	13.61(7.08,19.80)	13.95(7.65,21.25)	2.52(−17.33,34.37)	0.82(−0.19,1.83)
High-income Asia Pacific	6.72(2.09,11.98)	2.77(1.18,4.44)	1.00(0.42,1.66)	−64.03(−68.66,-54.30)	−3.80(−5.28,-2.30)
High-income North America	44.70(28.22,58.03)	3.90(2.73,5.02)	6.81(4.69,8.59)	74.38(56.86,97.48)	1.64(1.12,2.16)
North Africa and the Middle East	85.25(57.66,110.09)	26.35(15.88,36.47)	23.20(14.02,31.44)	−11.93(−29.15,10.97)	−0.06(−0.32,0.21)
Oceania	0.68(0.48,1.00)	11.36(7.24,16.17)	9.27(6.25,13.18)	−18.44(−35.19,9.27)	−0.70(−0.79,-0.61)
South Asia	61.43(40.11,90.56)	3.03(1.56,4.88)	4.73(2.81,7.34)	56.00(14.00,143.78)	1.44(1.26,1.63)
Southeast Asia	43.05(29.95,57.02)	5.70(3.36,8.20)	6.97(4.62,9.60)	22.34(0.20,61.45)	0.27(−0.20,0.75)
Southern Latin America	7.12(3.48,10.12)	8.15(4.96,11.09)	7.75(3.88,10.95)	−4.97(−26.41,6.16)	0.09(−0.12,0.30)
Southern Sub-Saharan Africa	13.65(9.93,17.28)	18.43(11.70,25.51)	28.40(17.52,37.54)	54.12(32.62,76.79)	1.56(1.08,2.03)
Tropical Latin America	17.13(10.98,22.65)	10.36(7.02,13.75)	6.90(4.29,9.26)	−33.40(−42.25,-25.23)	−0.90(−1.25,-0.55)
Western Europe	52.47(16.49,84.21)	3.94(1.93,5.81)	4.11(1.50,6.37)	4.28(−25.80,17.04)	0.21(−0.46,0.89)
Western Sub-Saharan Africa	22.55(13.46,30.63)	11.76(7.05,17.59)	13.03(7.79,18.62)	10.81(−26.50,48.36)	0.31(−0.02,0.65)

ASR, age-standardized rate per 100,000 population; BMI, body mass index; EAPC, estimated annual percentage change; GBD, Global Burden of Disease; HHD, hypertensive heart disease; PC, percentage change; UI, uncertainty interval.

### SDI-based trends

In high SDI regions, there was a notable increase in ASDR per 100,000 from 79.52 to 97.25 (EAPC: 1.16%). Conversely, high-middle SDI and middle SDI regions showed a decrease (EAPC: −0.11% and −0.60%, respectively), while low-middle SDI and low-SDI regions experienced an increase in ASDR (EAPC: 0.39 and 0.10%, respectively; Table 1; Figure 1A).

In high SDI regions, the ASMR rose from 3.88 to 4.38 per 100,000 (EAPC: 0.41%). High-middle SDI regions saw a decrease in the mortality rate, with AMR decreasing from 6.62 to 7.00 per 100,000 (EAPC: −0.35%). However, in low-middle SDI, the percentage increase was 10.41% (EAPC = 1.35%), and in low-SDI regions, the mortality rate increased with a rising ASMR, from 10.04 to 11.27 per 100,000 (EAPC: 0.40%; Table 2; Figure 1B).

### Regional trends

Regions with significant increases in DALYs included high-income North America, which saw a 79.40% (EAPC = 2.31%) increase; South Asia, 49.19% (EAPC = 1.41%) increase; Eastern Europe, which saw a 48.49% rise in the ASR for DALYs (EAPC: 1.05%); and Southern Sub-Saharan Africa, which experienced a 40.30% increase in DALYs (EAPC: 1.27%). Additionally, Central Sub-Saharan Africa reported a 39.93% increase in DALYs (EAPC: 1.03%; Table 1; Figure 1A). On the other hand, regions with significant declines in DALYs included Andean Latin America, with a 30.31% decrease in DALYs (EAPC: −0.59%); the highest decline was recorded in high-income Asia Pacific with −61.46% (EAPC = −2.84); and East Asia, which saw a 33.29% decrease in DALYs (EAPC: −1.38%). Central Latin America and Tropical Latin America both showed a 38% reduction in DALYs (EAPC: −1.70%; Table 1; Figure 1A).

Among GBD regions, high-income areas showed significant improvements. In high-income Asia Pacific, deaths from HHD due to high BMI dropped substantially by 64.03%, with an EAPC of −3.80%. Conversely, Central Sub-Saharan Africa experienced a 51.93% (EAPC = 1.52%) increase in mortality (EAPC: 1.95%), while Eastern Europe saw the most substantial increase in mortality with a 79.71% rise (EAPC: 1.43%). Other regions, such as high-income North America and South Asia, also reported notable increases in deaths, with high-income North America experiencing a 74.38% rise (EAPC: 1.64%) and South Asia showing a 56.00% increase (EAPC: 1.44%; Table 2; Figure 1B).

### National trends

**Figure 2:**
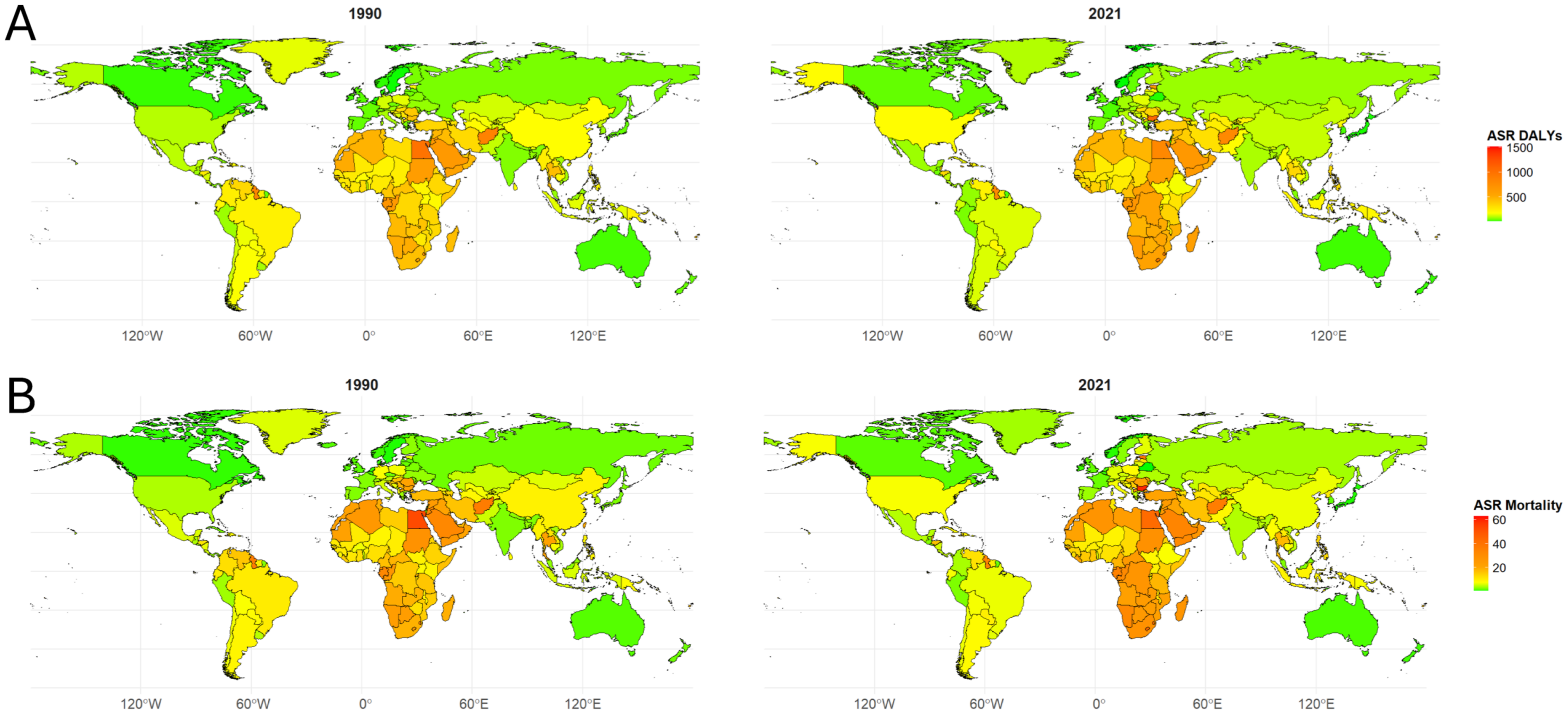
Global distribution of age-standardized disability-adjusted life year rates (ASDR) **(A)** and age-standardized mortality rates (ASMR) **(B)** per 100,000 population attributable to hypertensive heart disease (HHD) due to high body mass index (BMI) in 1990 and 2021. Color gradients represent the magnitude of rates across countries and regions, with darker shades indicating higher burden.

In 2021, the lowest burden of HHD due to high BMI was observed in Belarus, Japan, and Norway. Belarus saw a 73.08% decrease in ASMR and a 73.74% reduction in ASDR, with EAPC values of −5.33% and −5.40%, respectively. Japan followed with a 66.79% drop in ASMR and a 62.18% reduction in ASDR, reflecting the effectiveness of public health strategies. Norway experienced more modest declines, with a 23.04% decrease in ASMR and a 32.07% decrease in ASDR (Supplementary Table 1; Figures 2A,B).

Conversely, Bulgaria, Lesotho, and Egypt faced the highest burden. Bulgaria saw a 177.63% increase in ASMR and a 152.14% rise in ASDR, with EAPC values of 4.28 and 3.84%, indicating a sharp rise in the disease burden. Lesotho reported a 68.20% increase in ASMR and a 74.92% increase in ASDR, with EAPC values of 2.57 and 2.69%, reflecting growing challenges in managing HHD. Egypt also experienced a decline in both ASMR and ASDR but still faced a relatively high burden compared to other countries (Supplementary Table 1; Figures 2A,B).

### Gender trends

**Figure 3:**
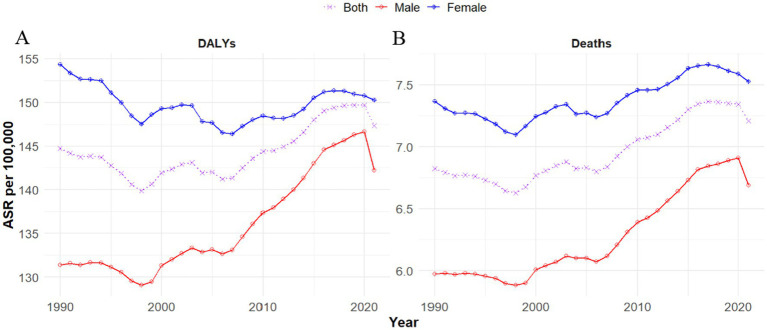
Global trends in age-standardized disability-adjusted life year rates (ASDR) **(A)** and age-standardized mortality rates (ASMR) **(B)** per 100,000 population attributable to hypertensive heart disease (HHD) due to high body mass index (BMI), stratified by sex, from 1990 to 2021.

Globally, there are notable gender differences in the burden of HHD attributable to high BMI. In 1990, men experienced 2,414.65 thousand DALYs, with an ASDR of 131.37 per 100,000. By 2021, this number rose to 5,646.14 thousand DALYs, with an ASDR of 142.25 per 100,000 (an EAPC 0.40%). Conversely, the DALYs for women increased from 3,251.37 thousand in 1990 to 6,905.61 thousand in 2021, with a slight decrease in the ASDR, from 154.37 per 100,000 to 150.26 per 100,000 (EAPC: –0.04) change (Table 1; Figure 3).

Globally, men and women experienced different trends in mortality due to HHD linked to high BMI. In 1990, men had an ASMR of 5.97 per 100,000. By 2021, male deaths increased to 243.06 thousand, with an ASMR of 6.69 per 100,000 (EAPC: 0.55%). For women, deaths increased from 146.48 thousand in 1990 to 351.84 thousand in 2021. The ASMR for women rose slightly from 7.37 per 100,000 to 7.53 per 100,000 (EAPC: 0.20%) (Table 2; Figure 3).

### Age-wise trend

**Figure 4:**
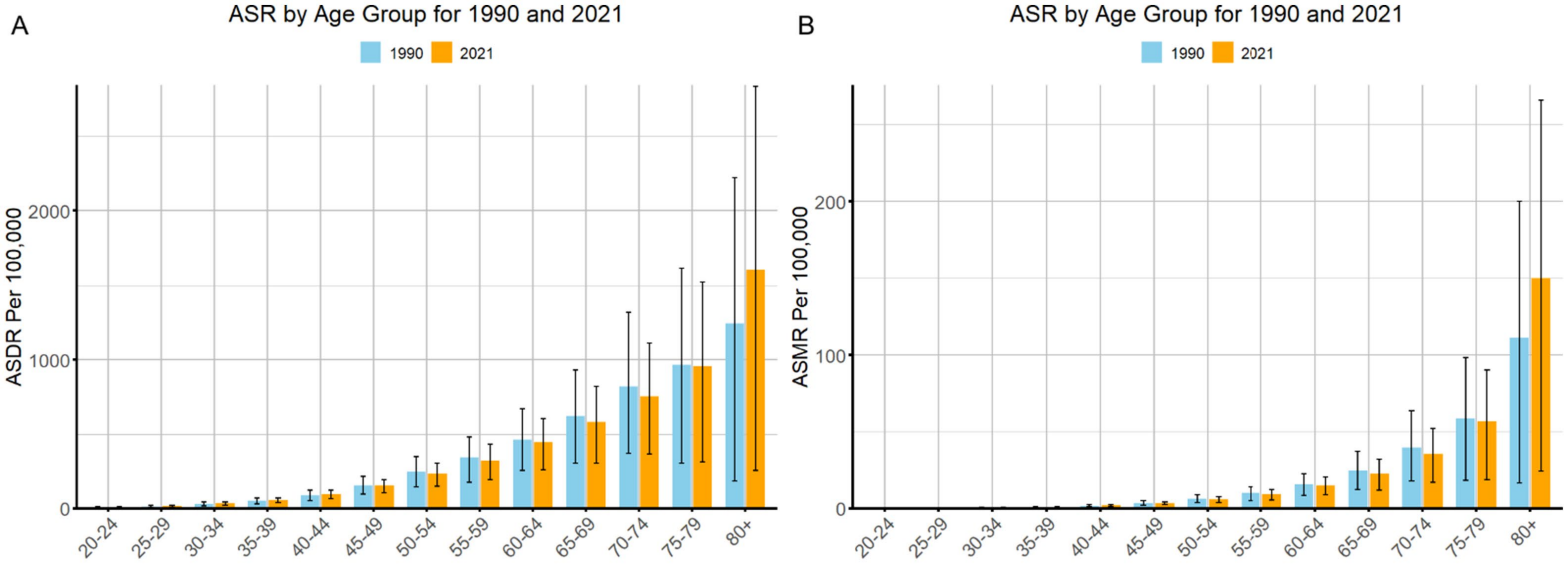
Global age-standardized disability-adjusted life year rates (ASDR) **(A)** and age-standardized mortality rates (ASMR) **(B)** per 100,000 population with 95% uncertainty intervals, attributable to hypertensive heart disease (HHD) due to high body mass index (BMI), stratified by age group, for the years 1990 and 2021.

The DALYs due to HHD followed a similar age group pattern in 2021. Younger age groups saw negligible or no burden, with ASR values of 0 for those under 20. The ASR increased with age, showing a gradual rise from the 20–24 to the 75–79 age groups. The 80 + age group experienced the highest ASR, rising sharply from 1243.80 in 1990 to 1604.32 in 2021 (Figure 4A).

In 2021, the global mortality of HHD due to high BMI showed a clear increase with age. The younger age groups (under 20) had no significant burden, with an ASR of 0 for age groups <5, 5–9, 10–14, and 15–19. The burden began to rise gradually in adults, with the ASR increasing steadily from 20–24 to 80+. While the 40–44 age group saw moderate increases, older age groups, especially those 80+, exhibited the sharpest rise, from 111.14 in 1990 to 150.01 in 2021 (Figure 4B).

### ARIMA forecast of ASDR and ASMR (2022–2035)

**Figure 5:**
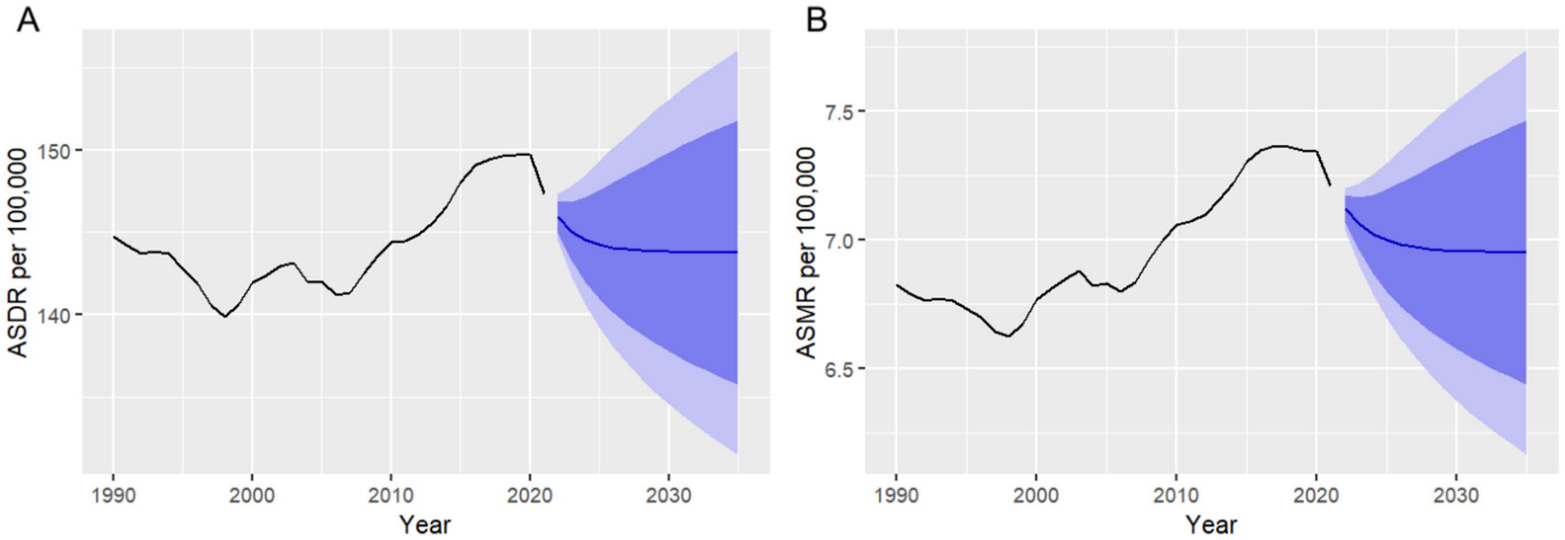
Autoregressive integrated moving average (ARIMA) model forecasts of global age-standardized rates (ASR) per 100,000 population for hypertensive heart disease (HHD) attributable to high body mass index (BMI), including age-standardized disability-adjusted life year rates (ASDR) **(A)** and age-standardized mortality rates (ASMR) **(B)**, projected from 2022 to 2035.

The ARIMA model provided annual forecasts for the global burden of HHD attributed to high BMI, projecting both the ASDR and ASMR from 2022 to 2035. For DALYs, the ASDR is expected to decrease gradually, from 145.91 per 100,000 in 2022 to 143.79 per 100,000 in 2035. While the reduction appears small, it reflects the ongoing burden of disability and premature mortality associated with HHD due to high BMI (Table 3; Figure 5A). In terms of mortality, the ASMR is forecasted to decrease slightly from 7.12 per 100,000 in 2022 to 6.95 per 100,000 by 2035. Although the reduction is modest, it indicates that HHD related to high BMI will continue to be a significant public health concern (Table 3; Figure 5B).

**Table 3:** Global projections of age-standardized disability burden and mortality from hypertensive heart disease attributable to high body mass index, 2022–2035.

Year	ASDR		ASMR	
	Forecast	95% CI	Forecast	95% CI
2022	145.91	144.47, 147.36	7.12	7.04, 7.2
2023	145.06	142.33, 147.78	7.06	6.9, 7.22
2024	144.55	140.62, 148.48	7.02	6.79, 7.26
2025	144.24	139.21, 149.27	7	6.7, 7.3
2026	144.06	138.03, 150.09	6.98	6.62, 7.35
2027	143.95	137.01, 150.89	6.97	6.54, 7.4
2028	143.88	136.11, 151.66	6.96	6.48, 7.45
2029	143.84	135.3, 152.39	6.96	6.43, 7.49
2030	143.82	134.56, 153.08	6.96	6.37, 7.54
2031	143.81	133.88, 153.73	6.96	6.33, 7.58
2032	143.8	133.24, 154.35	6.95	6.28, 7.62
2033	143.79	132.64, 154.94	6.95	6.24, 7.66
2034	143.79	132.07, 155.51	6.95	6.2, 7.7
2035	143.79	131.53, 156.05	6.95	6.17, 7.74

ARIMA, autoregressive integrated moving average model; ASDR, age-standardized disability-adjusted life years rate; ASMR, age-standardized mortality rate; BMI, body mass index; HHD, hypertensive heart disease.

## Discussion

This study provides a comprehensive analysis of the global, regional, and national trends in HHD attributable to high BMI over the past 30 years, using data from the GBD 2021. Our findings highlight that the global burden has risen substantially from 1990 to 2021. The total burden, measured in DALYs and deaths, has increased significantly, particularly due to the global rise in obesity. While ASR for both DALYs and mortality have shown slight increases, the overall impact of HHD has grown due to larger population sizes and the increasing prevalence of high BMI (27). The steady increase in DALYs and deaths underscores the growing global public health concern related to obesity and its cardiovascular risks. High BMI and hypertension are major upstream risk factors for hypertensive heart disease. The two constantly create a condition that seems to burden the system and the muscles. Even before a person becomes sick, these factors can cause minor damage to the heart’s structure and function. Therefore, a strategy for early detection must be developed (28).

SDI-based trends show that high SDI regions saw modest increases in both DALYs and deaths, with the ASR for DALYs increasing by 22.29% and a small rise in mortality rates. Conversely, low-SDI regions faced larger increases in both DALYs and deaths, highlighting a significant challenge in controlling obesity and its cardiovascular impacts in lower-income regions. Middle SDI regions exhibited mixed trends, with some improving while others faced rising burdens, indicating disparities in healthcare infrastructure and obesity management. Governments in low-SDI countries allocate a smaller share of their gross domestic product to healthcare systems in comparison to high SDI countries (2% vs. 12%, respectively) (29). Moreover, a low disease awareness rate (25–38%) has contributed to increased mortality rates in developing countries (30, 31). Enhancing global awareness of HHD could significantly improve the effectiveness of prevention, screening, and treatment efforts. Given the higher mortality in low-SDI countries, an integrated strategy encompassing education, health, and income initiatives is urgently needed to reduce the HHD burden (9). The higher burden and slower improvement in lower SDI regions may be linked to limited healthcare access, lower awareness, and fewer preventive measures targeting obesity and hypertension.

Region-wise trends show that North America and Eastern Europe experienced the largest increase in DALYs. On the other hand, Andean Latin America and East Asia experienced a decrease. This reflects regional disparities, with high-income regions bearing a greater burden, while low-income regions also continue to face rising challenges.

Earlier evidence suggests that high SDI regions experience a lower absolute burden of disease; the rising trend in these areas remains a concern. Projections suggest that such regions have a greater capacity to achieve health improvements. For instance, in high-income North America, the availability of advanced healthcare systems and lower population growth contribute to comparatively low absolute disease burden and increasing rates (32).

In high-income regions, the implementation of pharmacological and surgical interventions, like glucagon-like peptide-1 (GLP-1) receptor agonists and metabolic surgery, for their potential to reduce body weight, improve blood pressure control, and lower overall cardiometabolic risk, which are relevant to the prevention of hypertensive heart disease in obese populations, is still limited by ongoing concerns regarding cost and long-term safety. Insurance-related barriers add to these challenges; in the United States, for example, only a small proportion of commercial health plans, around 11%, provide coverage for weight loss medications, and limited reimbursement remains a major obstacle (33).

In 2021, the country-wise trends in HHD burden due to high BMI show that the lowest burden was observed in Belarus, Japan, and Norway, where both ASMR and ASDR showed significant declines. Belarus experienced the most substantial decrease, indicating a successful approach in managing the burden of HHD, likely due to public health interventions and improvements in healthcare access. Similarly, Japan reflects the effectiveness of its health strategies and the country’s strong healthcare infrastructure. Norway also showed improvements, suggesting that ongoing health initiatives have helped manage HHD, though at a more modest rate than in Belarus and Japan.

Conversely, regions such as Bulgaria, Lesotho, and Egypt faced the highest burden of HHD due to high BMI. Bulgaria saw a dramatic rise in both ASMR and ASDR, signaling a significant escalation in the disease burden, likely influenced by rising obesity rates and insufficient healthcare interventions. Lesotho also experienced an increase, highlighting growing challenges in managing the disease, which may be attributed to limited healthcare resources and rising obesity prevalence. Similarly, Egypt showed a substantial increase in both ASMR and ASDR, pointing to the increasing public health burden of HHD, driven by both the obesity epidemic and restricted healthcare access. Differences in socioeconomic development, obesity prevalence, and healthcare access may explain the contrasting national patterns. The sizeable increase in Bulgaria and Lesotho may be due to rising BMI, poor blood pressure control, and limited cardiovascular resources. Japan and Norway, on the other hand, benefited from strong prophylactic health systems, wise food choices, and early intervention of risk factors.

Marked regional disparities in the prevalence and mortality of HHD can largely be explained by three key factors. First, variations in healthcare infrastructure result in unequal outcomes for hypertension control and HHD management (9). Second, economic conditions shape both health behaviors and access to medical care, with populations in lower-income settings more vulnerable to unhealthy diets and lifestyles (34). Third, differences in public health strategies and levels of health literacy influence the success of prevention and management efforts (35). Collectively, these elements help explain the substantial global variation in HHD burden.

These trends clearly indicate the existence of a vast difference in the globally distributed burden of hypertensive heart disease attributable to the high BMI. Although higher-income states such as Belarus, Japan, and Norway have observed a decline in disease burden, lower-income states such as Bulgaria, Lesotho, and Egypt have already experienced an increase in burden due to limited resources and facilities, as adult obesity has risen. The above findings underscore the necessity of specific public health measures as well as healthcare-related interventions, especially in low- and middle-income areas, to mitigate the current burden of HHD, which has arisen as a result of high BMI.

Gender differences in the burden of hypertensive heart disease by high BMI indicate that both genders have been affected by increasing burden; however, the male gender has shown a more significant rise in the total burden of HHD. The ASRs for women, however, demonstrated an insignificant change; on the contrary, and in general, the burden of HHD among women has not increased as significantly as that of men has. These tendencies indicate that measures related to healthcare and population health might be affecting women to a greater extent than men. However, more efforts should be made to minimize their effects on both sexes.

As previous research has indicated similar findings in high SDI areas, excess body weight is found to be contributing to hypertensive heart disease in women to a larger extent. This is congruent with the evidence that females are more susceptible to the cardiometabolic implications of abdominal fat and a higher prevalence of diastolic dysfunction (36, 37). Biological mechanisms, such as sex-related differences in metabolic inflammation and insulin resistance, may contribute to this pattern (38). Conversely, in low- and lower-middle SDI regions, men experience a greater burden from lead exposure (an increase of 37.94% vs. 33.75% in men and women, respectively) and heavy alcohol consumption, reflecting occupational and lifestyle risks that are more common in disadvantaged populations (39, 40). Across all groups, high sodium intake consistently ranked as a leading contributor, with men bearing a larger share of the burden, likely due to persistent differences between sexes and regions in dietary habits and sodium-related cardiovascular risk (41, 42). These findings highlight how socio-demographic factors shape unequal exposure to metabolic, behavioral, and environmental risks, leading to wide variation in hypertensive heart disease burden worldwide.

The difference in the burden of hypertensive heart disease in men and women may be partially attributed to hormonal features, especially the decrease in the level of estrogens during the life course of women. Women of reproductive age tend to have lower blood pressure and a healthier cardiometabolic physiology, and this has been explained by the protective role of estrogen on endothelial activity, vascular tone, lipid metabolism, and inflammatory mediators (43, 44). The postmenopausal loss of estrogen levels is also associated with an increase in central adiposity, insulin resistance, and blood pressure, which can worsen the already adverse cardiovascular impact of high BMI and predispose people to hypertensive heart disease (45, 46). Hormonal changes can support the explanation of how sex differences in cardiovascular burden may be attenuated or reversed in old age and emphasize the need to use age- and sex-specific prevention methods.

The age-wise trends for HHD due to high BMI show a marked increase in burden with age. Younger age groups (<20 years) showed negligible or no significant burden, with ASR values remaining at 0 for these groups. Conversely, older age groups experienced significant rises in the ASR, with the 80 + age group seeing the largest increase, reflecting both aging populations and the growing impact of obesity-related cardiovascular diseases in older adults. This pattern highlights the need for targeted interventions for older adults and for preventing obesity at younger ages to reduce future health burdens.

We may explain these age and gender differences through a mix of biological and behavioral aspects. Some of these include sex-linked differences in fat distribution, hormones guarding against disease in premenopausal women, the higher obesity and high blood pressure in men in many areas, and sociocultural factors affecting access to healthcare and healthy lifestyle behaviors.

Prior research has similarly shown that the burden of disease is concentrated primarily among older adults. Advancing age amplifies the cardiovascular risks associated with obesity, largely through processes such as oxidative stress, chronic inflammation, and metabolic dysfunction. Furthermore, older patients frequently develop age-related structural and functional changes in the heart. They are disproportionately affected by chronic comorbidities, both of which complicate their clinical presentation and worsen their prognosis (47).

The dynamic ARIMA model predicts a slight decrease in the burden of HHD with high BMI between 2022 and 2035. It is anticipated that the ASDR and ASMR will decline slightly in 2035. Notwithstanding these cuts, the total burden remains high due to the rising obesity and population growth. Such forecasts underscore the necessity of improving these trends through further obesity prevention and specific cardiovascular health promotion programs.

The increased HHD associated with high BMI is another indication to prioritize obesity prevention and management policies worldwide. High SDI areas already benefit from public health efforts, health care infrastructure, and obesity-prevention information, but low-SDI areas need greater investments in health care facilities, lifestyle intervention programs, and policy-influenced measures. Our study, through the application of ARIMA-based modeling, projects that the burden of obesity-related HHD will escalate further, especially in low- and middle-income countries in the absence of effective interventions. These findings have important policy implications and underscore the urgent need to strengthen obesity prevention programs, promote early cardiovascular screening, and improve resource allocation within healthcare systems to address future challenges of disease burden. The use of the recent GBD 2021 dataset in our study provides updated and comprehensive global estimates. It offers timely evidence to inform preventive strategies, guide policymaking, and support targeted interventions.

A major strength of this study is the comprehensive analysis of global, regional, and national trends in HHD burden due to high BMI over three decades, using GBD data. However, limitations include potential misclassification of BMI-related risk factors and variations in data quality across countries. Incomplete primary data from various LMICs and regional discrepancies may affect the estimates and reporting accuracy in low-SDI countries, despite robust statistical adjustments of GBD 2021. Additionally, statistical models’ assumptions may not completely capture real-world complexities. Furthermore, forecasts based on historical trends may be limited in their ability to account for unexpected future events or shifts. Future research should focus on the impact of obesity interventions and the role of emerging risk factors, such as metabolic dysfunction-associated fatty liver disease, in driving HHD burden.

## Conclusion

The global burden of HHD due to high BMI has shown significant increases from 1990 to 2021, particularly in older age groups and low-SDI regions. Despite small reductions in ASRs, the total burden has grown due to rising obesity rates and population increases. Country-specific trends reflect varying success in managing HHD, with high SDI countries showing more progress. ARIMA-based forecasts suggest a gradual decline in the future burden, though challenges remain, particularly in lower-income regions. Targeted interventions and policies to reduce obesity, promote healthy diets, and improve hypertension control remain essential, particularly in low- and middle SDI regions, to mitigate the growing global burden of HHD attributable to high BMI.

## Data Availability

Publicly available datasets were analyzed in this study. This data can be found at: the data for this study are available from the Global Burden of Disease Study 2021 at: https://ghdx.healthdata.org/gbd-2021.

## Footnotes

1
https://vizhub.healthdata.org/gbd-compare

2
http://ghdx.healthdata.org
