# Supplementary material for: Global, regional, and national trends in hypertensive heart disease burden due to high BMI: a 30-year analysis using GBD 2021 data with projections to 2035
Source: Front Public Health. 2026 Jan 30;14:1701954. doi: 10.3389/fpubh.2026.1701954 (PMC12900672; doi:10.3389/fpubh.2026.1701954)
Supplement: Supplementary file 1 [file Table_1.docx]

**Global, Regional, and National Trends in Hypertensive Heart Disease Burden Due to High BMI: A 30-Year Analysis Using GBD 2021 Data with Projections to 2035**

Muhammad Babar Khawar^1,2,#^, **Kaleem Maqsood^3,#^, Sang Rui**^1,2,#^**,** Javeria Malik^4,#^**, Ali Afzal^5,#^,** Azeem Saeed^6^, Farwa Liaqat^4^, Humera Naveed^4^, Akasha Fiaz^4^, Chatchai Muanprasat^7,*^, Jing Zhou^1,*^

^1^Health Management Center, Affiliated Hospital of Yangzhou University, Yangzhou, Jiangsu, China.

^2^School of Basic Medical Sciences & School of Public Health, Faculty of Medicine, Yangzhou University, Yangzhou, 225009, PR China.

^3^Department of Biology, Lahore Garrison University, Lahore, Pakistan.

^4^Institute of Zoology, University of the Punjab, Lahore 54590, Pakistan.

^5^Shenzhen Institute of Advanced Technology, Chinese Academy of Sciences, Shenzhen, China.

^6^Allama Iqbal Medical College/Jinnah Hospital, Lahore, Punjab, Pakistan.

^7^Chakri Naruebodindra Medical Institute, Faculty of Medicine Ramathibodi Hospital, Mahidol University, Bangpla, Bangplee, Samut Prakarn 10540, Thailand.

# These authors contributed equally to this work

***Correspondence to:**

**1-Prof. Jing Zhou:** Health Management Center, Affiliated Hospital of Yangzhou University, Yangzhou, Jiangsu, China; [090380@yzu.edu.cn](mailto:090380@yzu.edu.cn).

**2-Prof. Chatchai Muanprasat:** Chakri Naruebodindra Medical Institute, Faculty of Medicine Ramathibodi Hospital, Mahidol University, Bangpla, Bangplee, Samut Prakarn, 10540 Thailand; Email: [chatchai.mua@mahidol.ac.th](mailto:chatchai.mua@mahidol.ac.th).

Table S1: Age-Standardized Mortality Rate (ASMR) and Age-Standardized Disability-Adjusted Life Years (ASDR) of hypertensive heart disease per 100,000 population for different countries in 1990 and 2021, along with their percentage changes over time.

| countries | ASMR, per 100,000, 1990 | ASMR, per 100,000, 2021 | percentage changes,  %, 1990-2021 | EAPC 1990-2021 | ASDR, per 100,000, 1990 | ASDR, per 100,000, 2021 | Percentage changes,  %, 1990-2021 | EAPC 1990-2021 |
| --- | --- | --- | --- | --- | --- | --- | --- | --- |
| Afghanistan | 36.97(14.60,61.15) | 36.60(17.84,58.05) | -1.01(-29.31,51.98) | -0.12(-0.25,0.00) | 865.66(315.13,1466.02) | 802.84(387.04,1302.15) | -7.26(-32.85,42.41) | -0.37(-0.51,-0.23) |
| Albania | 7.50(3.95,11.64) | 5.79(2.45,9.91) | -22.71(-56.03,33.29) | -0.42(-0.67,-0.17) | 129.10(79.12,189.37) | 92.36(49.77,146.75) | -28.46(-56.22,22.65) | -0.65(-0.89,-0.41) |
| Algeria | 25.95(12.71,42.50) | 27.42(12.35,42.80) | 5.66(-24.28,46.48) | 0.57(0.41,0.74) | 482.37(289.02,728.78) | 459.09(268.54,676.13) | -4.83(-28.72,34.07) | 0.08(-0.05,0.20) |
| American Samoa | 11.23(7.39,15.15) | 8.89(6.26,11.59) | -20.79(-39.28,7.08) | -0.98(-1.26,-0.70) | 278.83(198.62,349.45) | 219.90(168.73,278.56) | -21.13(-39.73,5.33) | -1.00(-1.28,-0.72) |
| Andorra | 5.14(2.01,8.60) | 3.59(1.35,6.25) | -30.27(-56.65,17.20) | -0.74(-0.93,-0.54) | 79.97(42.73,125.59) | 53.72(28.77,87.03) | -32.82(-57.89,14.01) | -0.90(-1.09,-0.71) |
| Angola | 19.45(10.10,29.51) | 22.51(13.29,35.02) | 15.72(-22.40,95.63) | 0.19(0.07,0.31) | 453.90(244.09,676.16) | 480.53(299.48,700.29) | 5.87(-28.80,82.52) | -0.08(-0.19,0.02) |
| Antigua and Barbuda | 15.96(10.32,21.71) | 24.66(15.05,33.16) | 54.47(31.57,75.51) | 1.76(1.29,2.24) | 350.21(272.03,435.79) | 486.53(370.60,600.67) | 38.93(20.83,55.41) | 1.46(0.98,1.94) |
| Argentina | 8.97(5.51,12.09) | 8.31(4.25,11.72) | -7.35(-27.09,4.02) | 0.16(-0.02,0.34) | 176.22(132.80,220.16) | 139.87(95.04,180.37) | -20.63(-32.23,-12.53) | -0.44(-0.61,-0.27) |
| Armenia | 9.73(5.62,13.97) | 8.28(4.51,11.85) | -14.92(-40.49,18.18) | -0.27(-0.65,0.12) | 180.01(127.01,237.40) | 145.55(95.58,201.08) | -19.14(-42.42,13.55) | -0.34(-0.66,-0.02) |
| Australia | 1.55(0.78,2.29) | 1.36(0.62,1.95) | -11.67(-24.78,0.15) | -0.10(-0.53,0.33) | 27.30(18.79,36.45) | 24.76(16.42,31.56) | -9.29(-18.55,-0.38) | -0.05(-0.50,0.40) |
| Austria | 4.65(2.32,6.75) | 5.65(2.05,8.88) | 21.45(-13.90,40.32) | 1.59(1.21,1.98) | 79.21(52.20,104.29) | 78.65(40.80,113.33) | -0.71(-23.68,12.83) | 0.80(0.47,1.13) |
| Azerbaijan | 13.71(8.19,20.51) | 11.47(6.75,17.63) | -16.39(-48.56,33.53) | -0.21(-0.45,0.03) | 286.27(193.48,395.45) | 228.30(147.87,332.85) | -20.25(-49.63,26.63) | -0.55(-0.82,-0.28) |
| Bahamas | 28.12(20.15,36.02) | 37.10(24.71,50.31) | 31.91(3.41,61.71) | 1.12(0.86,1.39) | 680.09(540.23,820.54) | 851.26(632.91,1078.31) | 25.17(-1.21,55.68) | 0.98(0.76,1.20) |
| Bahrain | 18.32(9.90,26.57) | 15.32(7.41,23.37) | -16.36(-43.40,21.97) | -0.80(-1.10,-0.50) | 326.77(217.89,440.03) | 249.19(149.96,358.19) | -23.74(-45.70,11.90) | -1.24(-1.44,-1.04) |
| Bangladesh | 3.38(1.84,5.75) | 4.68(2.25,9.36) | 38.33(-21.47,153.46) | 1.21(0.96,1.46) | 73.92(42.80,113.70) | 96.18(53.84,183.83) | 30.12(-22.40,145.14) | 1.17(0.97,1.37) |
| Barbados | 8.77(5.43,12.06) | 9.71(5.54,13.91) | 10.73(-12.28,36.66) | 0.91(0.75,1.08) | 181.40(136.37,226.03) | 196.58(141.24,263.64) | 8.36(-15.17,34.61) | 0.82(0.66,0.98) |
| Belarus | 2.55(1.79,3.40) | 0.69(0.48,0.91) | -73.08(-79.95,-64.82) | -5.33(-6.50,-4.15) | 64.62(48.15,82.59) | 16.97(13.09,21.78) | -73.74(-80.25,-65.26) | -5.40(-6.59,-4.19) |
| Belgium | 1.14(0.50,1.79) | 1.05(0.44,1.64) | -7.34(-23.51,7.31) | -0.36(-0.98,0.26) | 17.94(10.72,25.17) | 15.90(9.08,21.91) | -11.39(-22.38,-0.81) | -0.43(-0.92,0.07) |
| Belize | 9.82(6.85,12.66) | 16.13(10.59,21.24) | 64.33(40.99,89.33) | 2.11(1.83,2.39) | 225.90(180.00,273.85) | 356.34(277.77,439.27) | 57.75(35.74,81.99) | 1.92(1.66,2.18) |
| Benin | 8.95(5.15,13.14) | 10.78(6.06,15.67) | 20.35(-24.37,82.80) | 0.61(0.49,0.73) | 225.10(133.59,314.81) | 258.90(152.65,365.51) | 15.02(-26.84,70.39) | 0.44(0.31,0.56) |
| Bermuda | 5.15(3.20,6.98) | 5.26(3.00,7.30) | 2.15(-15.86,27.08) | 0.47(-0.11,1.04) | 105.37(79.24,132.91) | 104.76(75.09,134.87) | -0.58(-15.95,22.94) | 0.40(-0.18,0.97) |
| Bhutan | 7.33(3.30,12.18) | 6.78(3.89,10.56) | -7.55(-36.38,52.29) | -0.28(-0.34,-0.22) | 166.95(78.95,258.70) | 139.12(88.89,204.39) | -16.67(-42.85,34.86) | -0.66(-0.70,-0.62) |
| Bolivia (Plurinational State of) | 7.50(2.98,12.02) | 7.32(3.35,11.52) | -2.42(-29.69,40.35) | -0.03(-0.07,0.01) | 160.07(68.94,244.42) | 144.71(79.27,216.13) | -9.60(-35.07,36.49) | -0.36(-0.40,-0.32) |
| Bosnia and Herzegovina | 6.75(4.27,10.19) | 7.73(3.94,12.11) | 14.49(-29.28,68.79) | 0.69(0.50,0.88) | 137.32(95.47,194.10) | 132.67(81.87,192.99) | -3.39(-37.22,42.08) | 0.09(-0.10,0.28) |
| Botswana | 23.62(14.36,38.48) | 25.32(14.94,36.56) | 7.22(-30.86,83.83) | 0.61(0.28,0.94) | 504.37(327.36,749.41) | 497.27(340.74,698.25) | -1.41(-36.55,68.84) | 0.29(-0.04,0.62) |
| Brazil | 10.41(7.07,13.84) | 6.83(4.26,9.14) | -34.39(-43.10,-26.12) | -1.20(-1.36,-1.05) | 232.92(184.52,283.37) | 142.20(109.04,173.05) | -38.95(-45.05,-32.30) | -1.54(-1.69,-1.40) |
| Brunei Darussalam | 5.30(2.90,8.32) | 4.58(2.53,6.96) | -13.49(-36.91,25.05) | -0.12(-0.35,0.12) | 110.82(73.20,154.36) | 94.64(65.00,131.26) | -14.60(-37.81,24.35) | -0.35(-0.55,-0.15) |
| Bulgaria | 21.51(13.33,29.43) | 59.71(34.53,83.60) | 177.63(126.27,233.96) | 4.28(3.46,5.10) | 429.90(324.17,535.84) | 1083.92(779.93,1396.41) | 152.14(107.87,204.25) | 3.84(3.07,4.60) |
| Burkina Faso | 7.06(3.98,11.01) | 10.01(5.18,16.63) | 41.78(-6.74,112.07) | 1.38(1.27,1.48) | 181.65(106.43,271.50) | 243.06(128.27,382.08) | 33.81(-11.77,98.99) | 1.13(1.04,1.22) |
| Burundi | 10.72(0.93,19.09) | 9.53(4.43,15.62) | -11.08(-43.57,355.22) | -0.89(-1.10,-0.67) | 241.56(23.78,415.99) | 204.86(100.92,302.88) | -15.19(-46.79,321.24) | -1.06(-1.29,-0.83) |
| CÃ´te d'Ivoire | 11.11(5.77,16.35) | 15.38(8.21,22.68) | 38.42(-11.54,161.00) | 1.06(0.80,1.32) | 263.42(146.90,380.78) | 360.60(204.47,520.59) | 36.89(-13.53,153.43) | 1.03(0.77,1.28) |
| Cabo Verde | 9.45(5.76,13.94) | 10.48(6.42,15.04) | 10.86(-25.03,59.37) | 0.09(-0.22,0.40) | 237.73(157.89,333.77) | 231.29(166.60,312.19) | -2.71(-33.11,40.49) | -0.32(-0.57,-0.08) |
| Cambodia | 7.47(3.38,11.98) | 7.53(3.89,11.72) | 0.75(-31.80,63.19) | 0.00(-0.16,0.16) | 189.06(89.07,298.81) | 178.51(95.73,265.98) | -5.58(-37.38,57.74) | -0.22(-0.36,-0.08) |
| Cameroon | 16.73(8.05,24.42) | 18.44(8.55,28.66) | 10.22(-30.04,82.21) | 0.27(0.07,0.47) | 399.08(207.46,557.69) | 422.29(204.91,637.60) | 5.82(-32.75,58.68) | 0.14(-0.08,0.35) |
| Canada | 1.01(0.59,1.41) | 1.66(0.99,2.19) | 63.18(48.00,81.69) | 1.97(1.55,2.39) | 22.46(17.28,27.82) | 40.78(32.40,48.52) | 81.55(67.65,99.35) | 2.38(1.98,2.79) |
| Central African Republic | 18.27(6.15,30.77) | 24.91(9.44,42.94) | 36.35(-3.78,93.05) | 0.97(0.90,1.03) | 424.77(141.46,714.90) | 560.91(216.52,923.31) | 32.05(-4.96,87.79) | 0.85(0.79,0.91) |
| Chad | 10.20(4.38,15.69) | 12.97(5.85,20.24) | 27.13(-15.05,80.85) | 0.68(0.43,0.92) | 247.18(112.72,363.56) | 312.25(151.22,476.80) | 26.32(-15.79,82.35) | 0.64(0.39,0.89) |
| Chile | 7.07(4.08,9.82) | 6.87(3.16,9.76) | -2.77(-26.05,9.83) | 0.28(-0.01,0.57) | 131.05(93.14,167.23) | 113.04(73.79,144.73) | -13.74(-27.71,-3.44) | -0.08(-0.31,0.16) |
| China | 9.66(4.72,14.65) | 6.90(3.14,11.62) | -28.59(-50.23,6.93) | -1.17(-1.60,-0.73) | 182.56(104.75,260.36) | 120.03(71.90,180.13) | -34.25(-51.26,0.25) | -1.46(-1.90,-1.01) |
| Colombia | 12.36(7.68,17.27) | 3.98(2.09,5.81) | -67.79(-76.22,-60.02) | -4.08(-4.31,-3.84) | 243.14(177.22,308.23) | 73.42(49.29,99.00) | -69.80(-76.00,-63.12) | -4.34(-4.61,-4.06) |
| Comoros | 17.34(7.81,27.37) | 17.80(8.76,29.31) | 2.64(-28.75,56.59) | -0.14(-0.34,0.06) | 382.23(182.03,603.73) | 369.91(208.35,574.37) | -3.22(-32.67,51.30) | -0.39(-0.61,-0.16) |
| Congo | 27.03(12.64,43.42) | 31.50(16.46,49.46) | 16.54(-17.40,86.57) | 0.24(0.11,0.37) | 641.02(297.12,994.06) | 676.86(383.18,1007.27) | 5.59(-25.44,71.02) | -0.12(-0.28,0.04) |
| Cook Islands | 62.96(44.14,83.76) | 36.45(24.19,48.43) | -42.12(-57.95,-20.07) | -1.78(-1.89,-1.68) | 1515.14(1136.80,1925.02) | 858.60(639.22,1119.65) | -43.33(-59.53,-17.96) | -1.79(-1.91,-1.66) |
| Costa Rica | 5.57(3.43,7.80) | 4.99(2.63,7.12) | -10.45(-29.77,4.45) | -1.41(-1.87,-0.94) | 111.47(82.76,141.78) | 91.11(62.22,118.03) | -18.27(-31.55,-6.35) | -1.66(-2.04,-1.29) |
| Croatia | 14.68(7.53,21.74) | 7.74(3.74,11.21) | -47.30(-59.31,-36.97) | -1.06(-1.52,-0.58) | 239.15(157.29,327.96) | 121.21(77.57,162.83) | -49.32(-58.13,-39.66) | -1.35(-1.80,-0.90) |
| Cuba | 2.46(1.82,3.23) | 7.84(5.06,10.72) | 218.72(156.47,274.19) | 4.32(4.11,4.53) | 62.73(51.24,75.25) | 165.76(125.45,207.12) | 164.26(121.33,209.92) | 3.65(3.46,3.84) |
| Cyprus | 10.91(3.11,22.10) | 7.20(2.32,11.94) | -34.01(-60.63,45.78) | -1.45(-1.82,-1.09) | 148.04(57.98,277.17) | 89.31(42.91,135.43) | -39.67(-61.06,24.56) | -1.76(-2.03,-1.49) |
| Czechia | 2.21(1.56,2.83) | 5.58(2.98,7.97) | 152.30(77.28,200.32) | 2.81(1.91,3.72) | 49.72(39.72,59.43) | 95.73(64.02,125.74) | 92.54(51.15,127.01) | 1.93(1.13,2.74) |
| Democratic People's Republic  of Korea | 6.67(3.09,11.57) | 10.34(5.43,17.02) | 55.09(3.39,166.67) | 1.65(1.53,1.77) | 119.56(65.15,194.38) | 177.24(109.72,267.15) | 48.24(-1.72,148.70) | 1.46(1.36,1.56) |
| Democratic Republic of the Congo | 15.15(6.72,24.07) | 27.04(13.58,43.96) | 78.48(22.38,181.88) | 1.92(1.84,1.99) | 336.79(147.90,529.69) | 551.64(301.35,855.44) | 63.79(14.29,164.36) | 1.62(1.55,1.69) |
| Denmark | 1.12(0.61,1.62) | 1.02(0.42,1.58) | -8.60(-30.60,7.12) | -0.27(-0.58,0.04) | 20.03(14.08,26.41) | 16.45(10.14,22.48) | -17.85(-31.26,-6.65) | -0.65(-0.91,-0.39) |
| Djibouti | 12.14(7.01,19.40) | 11.28(5.89,19.64) | -7.13(-37.25,37.73) | -0.45(-0.53,-0.37) | 265.61(162.94,405.27) | 237.15(141.92,378.77) | -10.72(-39.40,34.45) | -0.60(-0.68,-0.51) |
| Dominica | 30.71(18.93,41.73) | 30.76(18.20,42.31) | 0.15(-19.43,25.64) | 0.14(-0.03,0.31) | 603.83(431.62,764.14) | 611.43(437.42,811.42) | 1.26(-20.27,27.75) | 0.24(0.03,0.44) |
| Dominican Republic | 7.89(4.96,11.57) | 8.89(5.34,13.28) | 12.63(-20.06,50.48) | 0.94(0.72,1.16) | 180.87(132.97,245.52) | 207.60(142.35,288.03) | 14.78(-14.46,51.27) | 0.92(0.75,1.10) |
| Ecuador | 10.20(5.92,14.45) | 7.32(3.52,11.22) | -28.25(-49.44,-7.64) | 0.41(-0.48,1.30) | 207.77(154.75,266.87) | 128.42(82.05,185.69) | -38.19(-53.94,-17.83) | -0.12(-1.05,0.82) |
| Egypt | 52.77(28.54,82.63) | 42.72(25.00,60.52) | -19.05(-38.39,7.36) | -0.33(-0.50,-0.16) | 987.40(649.85,1419.28) | 806.63(564.79,1086.13) | -18.31(-38.14,6.24) | -0.43(-0.58,-0.29) |
| El Salvador | 3.65(2.30,5.12) | 3.05(1.71,4.54) | -16.43(-39.32,11.60) | -0.59(-0.82,-0.37) | 77.65(57.79,100.21) | 59.88(40.61,83.52) | -22.89(-41.50,-0.36) | -0.88(-1.14,-0.61) |
| Equatorial Guinea | 28.87(15.70,45.88) | 25.79(12.44,46.01) | -10.65(-46.26,66.11) | -0.65(-0.97,-0.32) | 679.97(357.64,1082.97) | 524.48(272.37,864.79) | -22.87(-53.64,47.54) | -1.17(-1.51,-0.83) |
| Eritrea | 13.87(6.27,21.98) | 14.36(7.24,23.70) | 3.57(-32.02,74.64) | 0.09(-0.03,0.21) | 323.37(145.70,498.41) | 306.46(165.12,452.80) | -5.23(-36.01,62.75) | -0.20(-0.34,-0.06) |
| Estonia | 6.72(4.86,8.42) | 38.87(19.96,54.60) | 478.48(270.10,637.79) | 8.19(7.04,9.35) | 182.32(146.17,217.51) | 631.73(413.55,825.35) | 246.49(150.82,346.96) | 5.88(4.88,6.90) |
| Eswatini | 36.14(20.35,52.03) | 42.48(22.43,64.28) | 17.56(-19.90,66.56) | 0.99(0.51,1.46) | 740.46(458.08,1018.61) | 906.55(483.63,1372.17) | 22.43(-19.22,77.34) | 1.05(0.50,1.60) |
| Ethiopia | 13.91(7.08,20.64) | 7.54(4.35,12.04) | -45.79(-63.55,-4.43) | -2.55(-2.77,-2.33) | 336.22(171.30,486.87) | 167.65(109.45,244.03) | -50.14(-66.17,-6.24) | -2.88(-3.11,-2.65) |
| Fiji | 17.73(12.32,24.05) | 16.60(10.87,22.68) | -6.37(-30.24,28.50) | -0.50(-0.68,-0.32) | 434.73(327.82,564.77) | 376.47(272.84,503.69) | -13.40(-37.26,21.82) | -0.67(-0.80,-0.55) |
| Finland | 3.04(1.66,4.43) | 6.49(2.76,9.65) | 113.24(56.53,147.32) | 3.60(2.84,4.36) | 54.42(37.45,72.14) | 103.06(61.97,137.64) | 89.35(56.86,115.29) | 3.33(2.66,4.01) |
| France | 2.69(1.05,4.27) | 2.00(0.70,3.18) | -25.55(-41.81,-12.33) | -0.86(-1.02,-0.70) | 42.74(23.91,62.48) | 32.28(17.86,46.61) | -24.49(-35.16,-13.72) | -0.83(-0.95,-0.70) |
| Gabon | 32.80(17.70,49.79) | 35.02(18.98,58.78) | 6.79(-30.02,59.67) | 0.06(-0.11,0.22) | 702.10(401.54,1018.62) | 691.05(395.41,1079.35) | -1.57(-35.15,49.58) | -0.23(-0.42,-0.05) |
| Gambia | 11.86(7.02,17.99) | 18.30(9.88,27.85) | 54.35(0.62,130.38) | 1.26(1.05,1.46) | 288.71(183.13,415.47) | 435.11(247.71,638.75) | 50.71(-1.02,121.97) | 1.15(0.91,1.38) |
| Georgia | 6.93(4.81,9.48) | 23.52(13.24,32.44) | 239.28(142.77,344.79) | 6.67(5.31,8.04) | 156.12(119.20,204.47) | 434.00(309.91,555.50) | 177.99(117.08,264.21) | 5.52(4.29,6.77) |
| Germany | 7.54(3.89,10.97) | 6.21(2.14,9.66) | -17.71(-47.05,-3.24) | 0.49(0.10,0.88) | 131.46(89.98,170.59) | 85.13(42.50,119.89) | -35.25(-53.27,-23.88) | -0.25(-0.61,0.10) |
| Ghana | 12.14(7.58,18.31) | 15.77(9.06,23.46) | 29.88(-17.97,110.70) | 0.55(0.30,0.80) | 314.69(195.30,453.43) | 366.62(221.46,517.49) | 16.50(-23.81,94.42) | 0.21(-0.04,0.46) |
| Greece | 3.58(1.51,5.61) | 4.92(1.93,7.37) | 37.47(17.40,57.40) | 1.76(1.19,2.34) | 53.98(31.28,76.47) | 71.42(41.05,97.73) | 32.32(16.68,47.56) | 1.67(1.11,2.23) |
| Greenland | 6.14(3.96,8.57) | 3.78(2.52,5.12) | -38.40(-56.29,-15.88) | -1.30(-1.53,-1.07) | 151.74(115.33,197.85) | 99.37(73.88,126.86) | -34.51(-54.34,-11.97) | -1.06(-1.32,-0.81) |
| Grenada | 11.47(8.47,14.82) | 17.43(11.71,23.22) | 52.03(24.12,83.61) | 1.86(1.59,2.13) | 284.07(224.11,344.96) | 371.21(286.67,452.73) | 30.68(8.81,55.98) | 1.43(1.09,1.77) |
| Guam | 23.59(13.21,32.99) | 6.85(5.25,10.48) | -70.96(-78.82,-39.87) | -3.91(-4.58,-3.23) | 529.34(326.25,687.17) | 207.24(164.56,300.50) | -60.85(-70.77,-24.51) | -2.99(-3.63,-2.35) |
| Guatemala | 4.10(2.45,5.60) | 2.68(1.38,3.81) | -34.66(-48.46,-24.39) | -0.76(-1.27,-0.25) | 80.06(60.12,98.89) | 46.77(31.47,61.39) | -41.58(-51.89,-32.13) | -1.28(-1.81,-0.74) |
| Guinea | 10.80(5.51,16.81) | 13.66(7.34,20.98) | 26.43(-23.52,110.70) | 0.77(0.57,0.97) | 270.08(142.18,407.76) | 330.78(177.17,481.93) | 22.48(-24.96,106.89) | 0.65(0.47,0.84) |
| Guinea-Bissau | 16.22(7.69,25.92) | 21.16(10.06,33.13) | 30.49(-13.52,94.91) | 0.84(0.65,1.03) | 423.62(199.53,651.63) | 525.19(257.42,810.74) | 23.98(-16.36,91.23) | 0.67(0.48,0.86) |
| Guyana | 36.07(25.43,47.33) | 33.71(23.00,46.64) | -6.56(-28.21,19.24) | 0.43(0.05,0.80) | 829.44(639.85,1034.87) | 794.66(576.88,1060.21) | -4.19(-27.10,24.05) | 0.56(0.14,0.97) |
| Haiti | 10.09(3.78,17.51) | 11.87(5.03,20.65) | 17.56(-17.92,73.49) | 0.64(0.58,0.70) | 249.46(91.48,427.27) | 286.98(130.74,467.14) | 15.04(-22.10,74.64) | 0.60(0.53,0.66) |
| Honduras | 11.36(7.02,17.91) | 16.44(9.86,25.21) | 44.77(12.40,88.01) | 1.34(1.14,1.53) | 248.90(181.90,353.06) | 326.55(235.11,450.74) | 31.20(1.93,70.66) | 1.02(0.87,1.16) |
| Hungary | 16.68(9.91,22.57) | 16.90(9.68,22.93) | 1.32(-11.47,15.57) | 1.03(0.53,1.54) | 316.77(236.73,389.90) | 310.20(229.91,384.36) | -2.07(-13.84,10.11) | 0.77(0.29,1.26) |
| Iceland | 1.59(0.78,2.34) | 1.68(0.70,2.53) | 5.40(-15.65,22.53) | 1.11(0.75,1.46) | 26.06(16.75,35.10) | 24.81(14.56,34.72) | -4.80(-19.01,9.12) | 0.71(0.41,1.01) |
| India | 2.61(1.33,4.35) | 4.35(2.56,6.74) | 66.76(17.69,179.57) | 1.89(1.74,2.05) | 60.29(33.67,92.22) | 93.42(63.88,132.15) | 54.96(10.22,151.43) | 1.57(1.48,1.67) |
| Indonesia | 5.96(2.95,9.28) | 9.29(5.22,13.91) | 55.85(16.20,124.21) | 1.55(1.41,1.68) | 149.87(78.02,221.57) | 216.25(129.28,304.59) | 44.29(10.00,103.46) | 1.31(1.14,1.47) |
| Iran (Islamic Republic of) | 16.20(9.72,24.60) | 16.48(9.17,22.89) | 1.70(-23.12,35.21) | 0.43(0.26,0.61) | 327.60(236.86,453.71) | 304.65(218.62,388.26) | -7.01(-28.14,23.65) | 0.15(-0.03,0.32) |
| Iraq | 21.25(11.52,31.00) | 20.54(11.11,28.94) | -3.32(-33.88,55.46) | -0.66(-0.84,-0.49) | 446.70(258.18,630.79) | 375.81(246.43,509.09) | -15.87(-42.64,42.77) | -1.06(-1.21,-0.90) |
| Ireland | 1.61(0.75,2.43) | 1.13(0.46,1.74) | -29.77(-42.86,-17.71) | -0.11(-0.38,0.15) | 25.69(15.95,34.84) | 17.78(10.26,24.43) | -30.78(-40.34,-22.15) | -0.27(-0.53,-0.01) |
| Israel | 2.62(1.29,3.90) | 1.22(0.50,1.86) | -53.62(-65.07,-46.87) | -2.52(-3.32,-1.72) | 42.12(26.77,57.36) | 18.97(11.24,26.23) | -54.95(-61.41,-49.49) | -2.52(-3.28,-1.74) |
| Italy | 5.76(2.73,8.83) | 7.32(2.57,11.66) | 27.07(-10.28,48.51) | 0.95(0.82,1.07) | 91.49(57.05,125.70) | 101.33(50.64,144.00) | 10.75(-13.66,26.12) | 0.41(0.29,0.53) |
| Jamaica | 17.74(11.51,24.49) | 17.39(11.35,24.15) | -1.94(-23.91,27.80) | 0.87(0.15,1.60) | 370.14(284.20,466.52) | 392.20(284.14,527.44) | 5.96(-17.58,39.35) | 0.96(0.34,1.59) |
| Japan | 2.53(1.03,4.17) | 0.84(0.36,1.35) | -66.79(-70.45,-62.23) | -3.14(-4.11,-2.17) | 41.05(24.36,59.93) | 15.53(10.15,21.43) | -62.18(-65.82,-56.91) | -2.91(-3.79,-2.02) |
| Jordan | 32.55(18.83,46.57) | 24.41(13.70,34.49) | -25.01(-47.29,8.48) | -1.04(-1.25,-0.82) | 645.69(442.13,873.01) | 441.04(297.66,576.11) | -31.69(-51.06,1.42) | -1.39(-1.62,-1.16) |
| Kazakhstan | 5.31(3.87,6.63) | 5.25(3.34,7.14) | -1.17(-29.36,27.04) | -1.13(-2.88,0.65) | 130.96(106.95,156.00) | 107.38(79.59,140.22) | -18.00(-37.99,7.70) | -1.87(-3.65,-0.05) |
| Kenya | 9.04(5.35,13.81) | 14.10(7.37,21.83) | 56.03(16.53,101.56) | 1.90(1.72,2.08) | 198.80(131.06,277.31) | 286.37(175.82,398.55) | 44.05(9.43,86.44) | 1.58(1.42,1.74) |
| Kiribati | 8.65(5.64,11.62) | 9.00(5.61,12.22) | 4.01(-23.17,46.59) | 0.09(0.07,0.11) | 240.57(158.43,311.83) | 234.11(154.43,313.76) | -2.68(-28.38,40.75) | -0.14(-0.17,-0.12) |
| Kuwait | 22.71(14.35,30.72) | 11.22(5.77,16.40) | -50.62(-63.53,-37.82) | -2.16(-2.51,-1.81) | 461.90(355.41,570.72) | 196.80(129.64,273.79) | -57.39(-67.42,-46.04) | -2.65(-3.00,-2.29) |
| Kyrgyzstan | 5.92(4.11,7.95) | 10.21(6.70,13.68) | 72.58(33.44,116.06) | 1.39(1.00,1.77) | 137.70(106.34,169.17) | 214.81(164.14,273.56) | 56.00(22.90,93.53) | 1.01(0.68,1.34) |
| Lao People's Democratic Republic | 11.12(3.92,19.27) | 10.27(5.73,15.24) | -7.63(-40.42,98.83) | -0.27(-0.34,-0.21) | 274.51(98.34,453.73) | 243.06(140.67,342.51) | -11.46(-43.75,111.89) | -0.36(-0.40,-0.33) |
| Latvia | 1.71(1.25,2.13) | 11.67(6.73,15.63) | 582.94(397.21,725.48) | 8.46(7.18,9.76) | 44.50(35.74,52.58) | 216.24(157.87,267.10) | 385.92(285.45,483.05) | 6.79(5.60,7.99) |
| Lebanon | 15.71(5.72,27.24) | 7.38(3.82,10.77) | -53.03(-72.26,11.43) | -2.77(-2.96,-2.58) | 304.81(117.82,493.96) | 129.90(86.09,173.96) | -57.38(-74.01,1.59) | -3.07(-3.30,-2.85) |
| Lesotho | 25.64(15.52,39.17) | 43.12(21.12,64.86) | 68.20(4.61,157.60) | 2.57(2.04,3.10) | 532.98(350.15,748.32) | 932.26(490.14,1361.03) | 74.92(2.82,165.35) | 2.69(2.14,3.24) |
| Liberia | 15.07(9.15,22.03) | 20.92(11.06,32.76) | 38.86(-15.99,121.10) | 0.99(0.83,1.14) | 375.89(234.69,533.42) | 503.34(274.68,757.18) | 33.91(-18.72,117.25) | 0.85(0.70,1.01) |
| Libya | 14.91(7.88,23.52) | 23.79(12.04,37.29) | 59.52(6.84,139.43) | 2.00(1.83,2.16) | 310.57(175.40,476.10) | 471.02(260.00,729.20) | 51.66(4.49,126.26) | 1.69(1.55,1.83) |
| Lithuania | 1.94(1.38,2.49) | 6.41(4.07,8.41) | 230.01(150.28,323.29) | 4.87(4.16,5.58) | 48.11(37.11,58.46) | 127.75(96.09,160.59) | 165.54(109.97,247.87) | 4.00(3.25,4.76) |
| Luxembourg | 2.82(1.24,4.32) | 2.70(1.01,4.10) | -4.26(-25.23,13.37) | 0.35(0.11,0.60) | 43.75(26.16,60.94) | 38.61(21.12,54.35) | -11.75(-27.29,0.91) | 0.11(-0.13,0.34) |
| Madagascar | 19.08(11.47,29.87) | 27.12(15.71,41.70) | 42.09(-1.75,101.17) | 1.05(0.97,1.12) | 439.07(268.65,637.95) | 592.02(375.91,866.64) | 34.84(-8.06,92.75) | 0.87(0.79,0.95) |
| Malawi | 9.42(3.95,15.21) | 12.76(6.40,19.95) | 35.50(0.63,89.40) | 0.75(0.60,0.91) | 212.26(94.88,325.72) | 280.95(150.50,410.59) | 32.36(-3.18,89.85) | 0.62(0.45,0.79) |
| Malaysia | 3.07(1.88,4.29) | 2.65(1.75,3.73) | -13.58(-39.45,34.97) | -1.13(-1.44,-0.81) | 74.94(49.46,98.69) | 60.16(44.76,76.29) | -19.71(-40.79,20.99) | -1.32(-1.62,-1.02) |
| Maldives | 4.78(1.64,7.95) | 2.93(1.86,4.46) | -38.80(-63.92,71.27) | -1.90(-2.13,-1.68) | 124.45(43.62,198.92) | 67.43(48.30,93.97) | -45.81(-68.08,66.48) | -2.32(-2.55,-2.09) |
| Mali | 10.53(3.99,16.41) | 10.15(4.41,15.68) | -3.69(-34.05,40.82) | -0.16(-0.23,-0.09) | 264.53(101.44,404.49) | 255.45(115.88,385.24) | -3.43(-33.33,42.82) | -0.16(-0.22,-0.09) |
| Malta | 2.76(1.36,4.12) | 2.69(1.17,4.03) | -2.49(-22.43,14.55) | 0.65(0.33,0.97) | 45.49(29.73,61.41) | 43.82(26.81,59.08) | -3.68(-17.61,12.08) | 0.62(0.34,0.91) |
| Marshall Islands | 22.75(13.53,32.93) | 21.05(12.69,30.30) | -7.48(-29.47,19.67) | -0.35(-0.44,-0.25) | 567.97(355.36,808.47) | 534.88(322.78,768.21) | -5.83(-29.20,22.96) | -0.29(-0.41,-0.17) |
| Mauritania | 22.05(12.25,33.48) | 21.39(11.12,34.38) | -2.99(-39.92,55.89) | -0.26(-0.36,-0.17) | 513.50(294.87,760.34) | 465.29(268.67,744.76) | -9.39(-43.71,51.88) | -0.49(-0.58,-0.39) |
| Mauritius | 16.22(11.70,21.52) | 12.73(8.26,17.58) | -21.52(-37.82,-4.61) | -0.49(-1.16,0.17) | 380.65(298.86,469.08) | 275.63(217.10,342.22) | -27.59(-37.76,-16.28) | -0.78(-1.48,-0.07) |
| Mexico | 6.13(3.21,8.95) | 4.50(2.39,6.26) | -26.60(-42.79,-12.43) | -0.77(-1.06,-0.48) | 108.14(75.04,142.49) | 80.47(53.12,107.55) | -25.58(-42.50,-10.72) | -0.78(-1.09,-0.46) |
| Micronesia (Federated States of) | 24.01(13.67,33.73) | 21.38(12.92,29.61) | -10.94(-35.98,26.65) | -0.43(-0.46,-0.39) | 629.33(359.42,889.36) | 540.42(344.13,744.75) | -14.13(-39.71,26.25) | -0.55(-0.58,-0.51) |
| Monaco | 3.02(1.30,5.03) | 3.67(1.49,5.98) | 21.26(-29.44,84.03) | 0.83(0.39,1.29) | 49.90(30.22,77.15) | 58.00(32.61,84.69) | 16.25(-27.73,71.83) | 0.69(0.26,1.11) |
| Mongolia | 6.40(3.70,10.06) | 4.49(2.40,7.17) | -29.91(-60.31,22.20) | -1.68(-1.93,-1.42) | 140.02(84.12,206.58) | 92.13(57.69,136.41) | -34.20(-62.02,13.48) | -1.85(-2.12,-1.58) |
| Montenegro | 4.96(2.71,7.34) | 7.56(3.79,11.73) | 52.45(1.73,118.77) | 1.52(1.31,1.73) | 94.67(63.42,133.17) | 121.75(75.15,179.32) | 28.61(-11.40,87.22) | 0.92(0.84,1.01) |
| Morocco | 21.98(11.61,34.85) | 25.18(13.48,39.38) | 14.58(-17.32,51.41) | 0.71(0.56,0.87) | 459.95(244.42,687.37) | 489.60(286.75,725.69) | 6.45(-22.94,47.51) | 0.44(0.30,0.58) |
| Mozambique | 14.87(8.03,21.87) | 22.43(11.81,37.13) | 50.83(1.23,113.43) | 1.78(1.58,1.99) | 328.29(181.94,475.07) | 487.20(276.82,751.69) | 48.40(3.32,104.79) | 1.72(1.52,1.92) |
| Myanmar | 9.49(3.44,15.29) | 7.70(3.61,12.09) | -18.87(-45.75,47.10) | -1.03(-1.15,-0.90) | 247.55(91.61,397.88) | 187.96(95.84,286.12) | -24.07(-48.83,37.44) | -1.24(-1.38,-1.11) |
| Namibia | 23.79(13.65,36.44) | 32.17(18.00,49.60) | 35.26(-12.25,107.43) | 0.79(0.44,1.15) | 509.16(332.71,721.10) | 631.26(398.28,920.96) | 23.98(-19.99,88.06) | 0.44(0.05,0.83) |
| Nauru | 23.76(11.59,35.25) | 22.32(11.34,33.87) | -6.05(-28.31,25.48) | -0.31(-0.58,-0.04) | 646.11(305.64,942.13) | 581.64(284.42,863.54) | -9.98(-31.32,21.47) | -0.45(-0.73,-0.17) |
| Nepal | 2.97(1.46,4.92) | 4.48(2.77,6.94) | 50.75(-2.62,161.64) | 1.64(1.22,2.06) | 73.40(37.09,116.24) | 102.71(68.67,147.04) | 39.94(-8.96,152.55) | 1.40(0.98,1.82) |
| Netherlands | 1.04(0.52,1.56) | 1.39(0.46,2.22) | 33.78(-13.38,58.46) | 1.37(1.07,1.67) | 17.69(11.80,23.84) | 19.59(10.00,28.49) | 10.78(-17.48,28.11) | 0.80(0.52,1.07) |
| New Zealand | 2.05(1.25,2.89) | 1.19(0.60,1.70) | -41.79(-53.89,-34.78) | -2.12(-2.46,-1.78) | 41.07(30.97,51.39) | 21.80(15.27,27.72) | -46.92(-53.19,-42.04) | -2.48(-2.90,-2.07) |
| Nicaragua | 6.69(4.00,9.51) | 5.91(3.37,8.61) | -11.61(-31.58,9.72) | -0.46(-0.66,-0.26) | 135.70(97.05,176.54) | 114.12(80.32,155.69) | -15.90(-33.31,3.53) | -0.69(-0.85,-0.52) |
| Niger | 9.05(2.87,15.23) | 9.35(3.07,15.41) | 3.37(-35.27,151.46) | -0.02(-0.19,0.15) | 218.02(77.50,350.87) | 226.05(76.82,358.20) | 3.68(-34.00,150.01) | -0.02(-0.18,0.14) |
| Nigeria | 12.04(6.91,18.32) | 11.76(6.60,17.55) | -2.32(-41.35,38.10) | -0.51(-0.74,-0.28) | 261.91(167.51,388.15) | 254.16(146.87,359.53) | -2.96(-41.98,38.49) | -0.51(-0.74,-0.28) |
| Niue | 15.97(10.06,22.37) | 14.57(9.22,20.04) | -8.74(-34.01,24.01) | -0.47(-0.52,-0.42) | 391.99(260.55,539.92) | 347.48(232.57,463.35) | -11.36(-37.28,24.17) | -0.60(-0.66,-0.54) |
| North Macedonia | 19.90(10.62,30.15) | 25.01(10.33,40.14) | 25.68(-17.76,84.39) | 0.89(0.45,1.34) | 343.38(213.57,483.49) | 368.00(205.71,552.64) | 7.17(-25.50,54.22) | 0.44(0.09,0.79) |
| Northern Mariana Islands | 4.76(3.07,6.65) | 4.47(2.88,5.83) | -6.21(-28.77,26.36) | -0.12(-0.26,0.02) | 118.00(81.21,159.46) | 103.33(79.20,126.22) | -12.44(-33.66,17.11) | -0.30(-0.41,-0.18) |
| Norway | 1.17(0.57,1.76) | 0.90(0.34,1.45) | -23.04(-41.97,-12.11) | -0.41(-1.00,0.19) | 19.45(12.77,26.28) | 13.21(7.13,19.13) | -32.07(-44.81,-23.73) | -0.82(-1.42,-0.21) |
| Oman | 21.46(12.17,34.92) | 26.71(15.22,37.67) | 24.46(-26.54,97.62) | 1.60(1.08,2.12) | 456.92(293.08,717.54) | 498.82(333.96,675.71) | 9.17(-37.60,78.38) | 1.11(0.72,1.50) |
| Pakistan | 5.48(2.84,8.53) | 8.93(5.27,13.88) | 63.15(25.15,125.26) | 1.49(1.16,1.82) | 116.96(65.32,170.76) | 185.96(121.14,272.14) | 58.99(22.93,118.57) | 1.40(1.05,1.75) |
| Palau | 6.36(4.39,8.88) | 5.54(3.81,7.60) | -12.88(-38.77,15.31) | -0.36(-0.42,-0.31) | 157.61(114.96,215.65) | 133.36(97.08,175.11) | -15.39(-42.37,14.93) | -0.51(-0.56,-0.46) |
| Palestine | 31.54(16.30,49.88) | 24.12(12.35,34.76) | -23.53(-44.28,18.51) | -0.82(-1.03,-0.61) | 572.30(347.31,864.12) | 403.91(260.58,538.63) | -29.42(-49.57,14.47) | -1.10(-1.25,-0.94) |
| Panama | 3.24(1.92,4.38) | 4.84(2.67,6.73) | 49.35(16.53,79.70) | 0.96(0.40,1.52) | 63.63(46.78,80.27) | 90.46(61.98,118.45) | 42.16(14.26,70.58) | 0.78(0.25,1.30) |
| Papua New Guinea | 8.00(4.12,12.76) | 7.40(4.09,12.49) | -7.40(-38.30,47.71) | -0.31(-0.35,-0.28) | 223.72(113.55,355.53) | 207.21(115.01,349.31) | -7.38(-39.46,50.65) | -0.31(-0.35,-0.27) |
| Paraguay | 8.19(4.96,11.76) | 10.02(5.70,14.42) | 22.36(-7.93,58.83) | 0.94(0.82,1.07) | 171.72(123.27,224.09) | 198.20(136.05,273.43) | 15.42(-12.86,50.63) | 0.63(0.51,0.75) |
| Peru | 3.69(2.16,5.42) | 2.56(1.37,3.92) | -30.57(-51.59,-3.57) | -1.41(-1.72,-1.10) | 80.85(57.35,105.81) | 54.81(37.35,77.92) | -32.20(-50.97,-7.21) | -1.44(-1.75,-1.14) |
| Philippines | 9.17(6.37,13.15) | 13.31(9.00,17.91) | 45.21(18.66,77.92) | 1.42(1.30,1.55) | 220.10(167.78,280.47) | 321.32(234.10,411.39) | 45.99(19.87,80.79) | 1.40(1.27,1.53) |
| Poland | 7.21(4.63,9.53) | 8.00(4.43,10.94) | 10.93(-11.12,25.26) | 0.24(-0.05,0.53) | 149.64(116.13,183.83) | 148.20(103.98,189.19) | -0.96(-15.10,11.18) | -0.06(-0.37,0.26) |
| Portugal | 3.57(1.50,5.62) | 3.19(1.07,5.06) | -10.62(-30.66,3.77) | -0.30(-0.48,-0.12) | 54.64(31.76,77.05) | 45.92(23.75,65.77) | -15.97(-28.55,-4.22) | -0.44(-0.59,-0.28) |
| Puerto Rico | 7.49(5.15,9.81) | 6.61(4.23,8.99) | -11.77(-28.13,4.53) | -0.06(-0.43,0.31) | 170.33(135.60,203.81) | 148.65(116.08,186.73) | -12.73(-27.10,2.68) | -0.14(-0.49,0.22) |
| Qatar | 18.31(9.56,26.81) | 9.18(4.68,13.45) | -49.83(-64.62,-30.96) | -2.61(-3.21,-2.02) | 314.03(198.67,424.03) | 154.21(96.21,215.63) | -50.89(-64.92,-31.57) | -2.52(-3.01,-2.03) |
| Republic of Korea | 4.46(1.90,7.27) | 1.89(0.56,3.59) | -57.62(-77.80,-12.25) | -2.71(-2.98,-2.44) | 79.27(44.68,115.41) | 27.47(12.46,46.46) | -65.34(-79.18,-20.64) | -3.41(-3.65,-3.16) |
| Republic of Moldova | 2.87(2.10,3.58) | 19.39(12.75,25.69) | 576.69(449.88,710.61) | 7.24(6.81,7.68) | 72.33(59.26,85.17) | 360.00(269.91,450.70) | 397.71(309.89,487.07) | 5.92(5.48,6.36) |
| Romania | 21.67(12.79,30.33) | 20.39(10.44,28.56) | -5.89(-22.23,9.06) | 0.44(0.05,0.83) | 394.62(285.14,501.63) | 350.94(237.75,451.91) | -11.07(-24.71,2.90) | 0.25(-0.13,0.62) |
| Russian Federation | 2.13(1.61,2.61) | 3.78(2.50,4.88) | 77.74(44.86,97.88) | 1.75(0.39,3.13) | 54.30(45.44,62.08) | 78.14(60.34,94.07) | 43.92(24.14,59.97) | 0.76(-0.56,2.10) |
| Rwanda | 18.59(4.43,29.77) | 12.44(3.35,21.62) | -33.04(-59.24,25.17) | -2.23(-2.62,-1.84) | 433.38(100.37,680.74) | 251.39(70.11,394.66) | -41.99(-64.14,13.08) | -2.80(-3.22,-2.37) |
| Saint Kitts and Nevis | 13.46(8.90,17.93) | 17.10(10.07,23.31) | 27.06(2.19,52.87) | 1.81(1.32,2.29) | 301.77(233.90,371.29) | 338.60(247.25,438.36) | 12.21(-10.24,35.61) | 1.30(0.77,1.84) |
| Saint Lucia | 17.96(10.55,25.73) | 15.87(8.99,22.88) | -11.60(-27.93,6.27) | -0.23(-0.78,0.32) | 365.70(277.28,464.93) | 322.64(236.46,418.56) | -11.78(-27.15,7.20) | -0.10(-0.62,0.43) |
| Saint Vincent and the Grenadines | 16.34(10.60,23.06) | 26.32(16.30,36.80) | 61.07(35.67,89.08) | 1.62(1.02,2.22) | 354.94(267.59,449.44) | 558.16(425.97,705.61) | 57.25(33.98,83.33) | 1.55(0.98,2.12) |
| Samoa | 19.88(12.46,27.84) | 17.16(10.82,23.51) | -13.66(-33.55,15.89) | -0.51(-0.64,-0.38) | 493.82(328.02,650.99) | 420.62(280.04,553.50) | -14.82(-35.34,17.23) | -0.54(-0.69,-0.39) |
| San Marino | 3.99(1.64,6.66) | 2.53(1.00,4.10) | -36.57(-59.45,-3.76) | -0.34(-0.76,0.08) | 60.06(32.76,92.04) | 41.00(21.78,63.73) | -31.73(-55.37,-0.31) | -0.12(-0.49,0.25) |
| Sao Tome and Principe | 7.75(5.21,10.55) | 9.20(5.89,13.23) | 18.62(-13.14,64.38) | 0.43(0.30,0.56) | 187.38(137.40,242.41) | 215.30(152.15,294.08) | 14.90(-15.97,58.87) | 0.23(0.04,0.43) |
| Saudi Arabia | 32.22(16.86,50.08) | 33.52(15.82,50.48) | 4.04(-31.39,56.84) | -0.07(-0.22,0.08) | 650.11(380.00,993.40) | 661.34(359.51,975.85) | 1.73(-33.30,55.73) | -0.08(-0.22,0.07) |
| Senegal | 10.84(6.96,15.62) | 14.19(8.05,20.96) | 30.95(-9.73,89.44) | 0.88(0.78,0.97) | 269.61(177.71,376.53) | 332.99(203.21,469.65) | 23.51(-14.28,78.98) | 0.72(0.61,0.82) |
| Serbia | 17.64(8.58,27.89) | 14.76(7.16,22.04) | -16.31(-43.77,20.61) | -0.89(-1.08,-0.70) | 282.04(172.23,413.72) | 234.09(143.24,325.33) | -17.00(-42.19,18.92) | -0.80(-0.96,-0.64) |
| Seychelles | 33.01(23.64,44.20) | 28.42(18.31,39.92) | -13.90(-32.64,6.35) | -0.44(-0.61,-0.28) | 785.90(600.07,959.33) | 594.82(454.14,769.85) | -24.31(-39.56,-5.77) | -0.88(-1.05,-0.70) |
| Sierra Leone | 8.81(5.07,13.66) | 12.06(6.27,18.47) | 36.93(-6.55,102.27) | 1.11(0.84,1.38) | 209.85(125.03,301.85) | 288.83(156.27,428.00) | 37.64(-4.09,103.44) | 1.16(0.89,1.42) |
| Singapore | 4.12(2.50,6.00) | 2.73(1.75,3.78) | -33.66(-44.64,-20.83) | -1.04(-1.45,-0.63) | 90.65(67.37,117.70) | 62.61(49.04,77.74) | -30.93(-40.48,-20.54) | -0.90(-1.32,-0.49) |
| Slovakia | 6.64(3.93,10.76) | 7.18(3.83,10.75) | 8.10(-39.30,74.41) | 0.81(0.53,1.08) | 137.44(95.03,201.50) | 132.58(86.26,186.50) | -3.53(-41.86,49.58) | 0.36(0.07,0.65) |
| Slovenia | 9.15(5.50,12.43) | 9.83(3.49,15.10) | 7.48(-40.98,31.37) | 0.94(0.63,1.25) | 171.39(125.12,214.40) | 135.55(69.70,193.57) | -20.91(-47.77,-3.92) | -0.24(-0.52,0.04) |
| Solomon Islands | 10.38(5.38,15.86) | 10.63(6.08,15.89) | 2.47(-26.54,47.69) | 0.10(0.05,0.15) | 274.25(135.06,423.29) | 282.24(168.71,424.63) | 2.91(-27.82,55.99) | 0.13(0.07,0.19) |
| Somalia | 16.59(7.68,25.43) | 15.98(6.94,26.04) | -3.67(-32.86,41.94) | -0.12(-0.25,0.00) | 406.58(185.25,640.04) | 368.41(166.02,567.92) | -9.39(-37.09,34.38) | -0.39(-0.53,-0.25) |
| South Africa | 18.67(11.76,26.97) | 28.15(17.06,38.09) | 50.75(27.23,75.27) | 1.38(0.89,1.89) | 425.86(326.62,555.64) | 569.58(422.84,712.96) | 33.75(17.76,51.87) | 1.02(0.54,1.50) |
| South Sudan | 9.56(4.12,15.76) | 8.73(3.58,13.77) | -8.65(-35.94,32.12) | -0.50(-0.87,-0.13) | 215.46(100.97,347.61) | 197.97(88.18,301.63) | -8.12(-35.35,38.22) | -0.50(-0.89,-0.10) |
| Spain | 2.65(1.13,4.02) | 3.52(1.26,5.37) | 32.85(6.10,49.12) | 1.08(0.90,1.26) | 40.25(23.24,56.17) | 49.46(25.76,69.01) | 22.90(5.26,36.07) | 0.81(0.67,0.95) |
| Sri Lanka | 8.00(4.98,11.58) | 5.97(3.23,10.68) | -25.37(-55.05,38.26) | -1.11(-1.32,-0.91) | 181.32(130.05,241.48) | 130.50(77.75,221.27) | -28.02(-56.56,35.56) | -1.33(-1.56,-1.10) |
| Sudan | 28.50(14.76,43.33) | 28.70(15.27,44.64) | 0.72(-30.01,50.20) | 0.04(-0.11,0.18) | 617.32(319.73,923.89) | 581.26(333.71,885.15) | -5.84(-35.48,44.06) | -0.17(-0.31,-0.04) |
| Suriname | 10.15(6.82,14.29) | 10.12(6.49,15.11) | -0.27(-26.30,30.76) | 0.29(0.11,0.47) | 238.46(179.72,307.23) | 240.71(171.15,326.63) | 0.94(-24.73,33.62) | 0.23(0.05,0.42) |
| Sweden | 0.83(0.38,1.29) | 2.90(0.98,4.68) | 248.39(144.37,313.91) | 5.53(4.91,6.16) | 14.12(8.56,19.74) | 41.57(19.93,60.42) | 194.36(134.14,239.58) | 4.99(4.39,5.59) |
| Switzerland | 3.62(1.51,5.63) | 3.99(1.41,6.59) | 10.27(-18.23,28.33) | 0.86(0.44,1.29) | 54.92(32.75,78.34) | 54.32(26.10,81.52) | -1.11(-21.62,13.45) | 0.46(0.11,0.81) |
| Syrian Arab Republic | 24.50(13.56,35.96) | 24.78(12.68,36.81) | 1.15(-32.74,47.31) | -0.42(-0.72,-0.11) | 468.53(308.28,630.82) | 429.61(262.54,614.01) | -8.31(-39.05,39.89) | -0.77(-1.11,-0.43) |
| TÃ¼rkiye | 19.91(9.41,30.03) | 15.21(6.84,22.63) | -23.58(-47.95,12.82) | -0.60(-1.08,-0.12) | 345.20(202.00,500.01) | 241.80(145.20,339.30) | -29.96(-52.25,7.95) | -1.06(-1.45,-0.68) |
| Taiwan (Province of China) | 7.18(4.30,10.67) | 4.92(2.52,7.23) | -31.49(-43.43,-20.37) | -0.03(-0.87,0.82) | 136.32(98.16,180.79) | 99.27(70.22,127.78) | -27.18(-36.89,-16.80) | 0.35(-0.56,1.26) |
| Tajikistan | 21.74(11.24,38.73) | 17.72(9.29,26.41) | -18.52(-49.19,36.08) | -0.91(-1.33,-0.48) | 439.07(281.04,667.89) | 340.78(219.36,484.00) | -22.39(-50.45,22.62) | -1.07(-1.44,-0.70) |
| Thailand | 1.14(0.64,1.77) | 1.33(0.82,2.04) | 16.56(-20.48,82.80) | 0.13(-0.03,0.29) | 27.14(17.74,37.87) | 31.81(22.91,44.35) | 17.22(-16.79,80.42) | 0.12(-0.07,0.30) |
| Timor-Leste | 4.88(2.10,8.37) | 5.81(3.43,8.97) | 19.14(-22.69,112.85) | 0.71(0.43,1.00) | 113.72(49.79,187.24) | 141.89(88.63,212.25) | 24.77(-22.75,153.12) | 0.87(0.56,1.17) |
| Togo | 11.49(6.63,18.04) | 17.18(8.93,26.42) | 49.58(-1.98,116.35) | 1.34(1.14,1.53) | 280.41(170.76,413.36) | 395.40(211.24,602.52) | 41.01(-6.75,104.57) | 1.15(0.95,1.35) |
| Tokelau | 18.55(11.93,26.58) | 13.15(8.28,18.69) | -29.11(-47.55,-3.09) | -1.20(-1.24,-1.16) | 467.94(309.28,654.69) | 322.83(214.51,453.87) | -31.01(-51.07,-1.97) | -1.32(-1.36,-1.27) |
| Tonga | 4.95(3.21,6.83) | 5.07(3.40,6.89) | 2.46(-27.30,43.11) | 0.06(-0.10,0.23) | 126.53(89.67,168.94) | 122.06(87.26,162.51) | -3.53(-33.04,34.83) | -0.15(-0.30,0.00) |
| Trinidad and Tobago | 19.13(12.10,26.99) | 10.90(7.07,15.42) | -43.01(-56.71,-26.58) | -2.00(-2.29,-1.71) | 398.18(308.59,493.52) | 256.31(189.83,340.45) | -35.63(-51.75,-17.35) | -1.68(-1.97,-1.40) |
| Tunisia | 17.47(9.79,26.77) | 21.10(9.45,39.42) | 20.72(-25.52,124.67) | 0.68(0.62,0.75) | 343.37(226.14,492.06) | 385.35(194.06,684.24) | 12.23(-31.52,112.85) | 0.43(0.37,0.48) |
| Turkmenistan | 8.68(5.15,12.50) | 11.39(6.98,17.47) | 31.25(-8.78,86.77) | 0.55(0.30,0.81) | 196.34(132.24,268.35) | 249.52(162.64,374.88) | 27.09(-11.66,82.70) | 0.50(0.24,0.75) |
| Tuvalu | 22.83(13.25,32.38) | 17.50(11.36,24.10) | -23.36(-42.93,12.32) | -0.83(-0.90,-0.76) | 601.11(352.28,835.25) | 437.90(297.01,591.49) | -27.15(-45.93,8.80) | -0.99(-1.07,-0.92) |
| Uganda | 8.32(2.55,14.09) | 9.71(3.49,17.74) | 16.65(-22.97,87.53) | 0.02(-0.18,0.22) | 188.89(60.26,307.97) | 208.77(83.95,334.95) | 10.53(-25.86,71.48) | -0.23(-0.46,0.00) |
| Ukraine | 3.31(2.31,4.33) | 4.28(2.67,6.15) | 29.17(-3.44,73.69) | 0.55(0.26,0.84) | 74.42(59.10,89.52) | 94.41(64.64,128.57) | 26.86(-6.93,71.96) | 0.52(0.25,0.80) |
| United Arab Emirates | 18.07(9.85,28.25) | 21.20(12.20,29.33) | 17.33(-22.07,66.65) | 2.43(1.72,3.15) | 364.92(233.00,531.80) | 369.47(251.20,491.63) | 1.25(-34.60,41.65) | 1.57(1.00,2.15) |
| United Kingdom | 1.65(1.10,2.16) | 1.86(1.10,2.48) | 12.56(-2.88,23.30) | 0.96(0.77,1.14) | 35.16(27.42,42.66) | 38.24(29.47,46.21) | 8.75(-0.01,17.96) | 0.86(0.69,1.03) |
| United Republic of Tanzania | 15.94(6.21,25.71) | 18.12(7.00,29.89) | 13.69(-19.43,60.98) | 0.41(0.34,0.49) | 337.26(154.51,504.20) | 353.84(153.42,540.51) | 4.91(-24.48,51.42) | 0.13(0.07,0.18) |
| United States of America | 4.20(2.95,5.38) | 7.44(5.13,9.40) | 77.26(58.76,101.10) | 2.09(1.94,2.25) | 103.83(84.32,122.70) | 187.98(155.90,219.82) | 81.05(62.32,104.53) | 2.34(2.20,2.49) |
| United States Virgin Islands | 17.00(10.55,24.20) | 9.75(5.48,14.34) | -42.66(-59.19,-19.50) | -1.39(-1.55,-1.23) | 353.58(262.54,470.06) | 207.53(142.96,286.96) | -41.31(-58.36,-16.30) | -1.32(-1.48,-1.16) |
| Uruguay | 4.55(2.82,6.19) | 6.47(3.34,9.17) | 42.02(10.55,60.76) | 1.05(0.92,1.18) | 88.86(66.18,110.88) | 110.70(76.54,141.88) | 24.57(6.50,37.59) | 0.67(0.51,0.83) |
| Uzbekistan | 7.27(4.54,10.84) | 11.34(7.19,16.28) | 55.92(3.25,128.54) | 1.69(1.43,1.95) | 167.48(112.61,235.82) | 241.96(167.97,334.49) | 44.47(-0.74,105.66) | 1.38(1.12,1.64) |
| Vanuatu | 11.54(6.63,17.17) | 12.44(7.97,17.10) | 7.78(-19.03,43.82) | 0.18(0.13,0.24) | 298.93(181.68,446.23) | 325.67(220.35,440.92) | 8.94(-21.49,47.99) | 0.19(0.12,0.27) |
| Venezuela (Bolivarian Republic of) | 15.98(10.78,21.44) | 11.96(6.53,17.62) | -25.11(-43.34,-4.45) | -1.30(-1.65,-0.96) | 334.77(256.40,411.60) | 228.02(157.99,323.83) | -31.89(-47.77,-13.32) | -1.65(-2.02,-1.28) |
| Viet Nam | 3.94(2.07,6.81) | 4.69(2.41,7.93) | 19.04(-25.88,88.81) | 0.87(0.65,1.08) | 87.59(53.07,141.90) | 100.48(57.12,158.84) | 14.71(-26.02,79.90) | 0.83(0.55,1.10) |
| Yemen | 25.45(12.14,41.07) | 31.93(15.18,54.51) | 25.46(-18.97,115.96) | 0.56(0.36,0.75) | 552.43(270.79,848.11) | 643.38(336.39,1081.30) | 16.46(-24.85,104.56) | 0.32(0.16,0.49) |
| Zambia | 18.38(10.73,26.58) | 24.44(12.11,37.63) | 32.95(-10.17,88.63) | 0.67(0.53,0.80) | 421.59(248.32,586.38) | 512.88(267.16,749.03) | 21.65(-20.09,75.60) | 0.31(0.17,0.46) |
| Zimbabwe | 12.07(7.63,18.72) | 23.98(14.67,36.18) | 98.60(26.45,179.12) | 3.00(2.44,3.56) | 261.99(183.01,371.58) | 540.18(338.44,796.76) | 106.19(28.75,189.61) | 3.13(2.51,3.76) |
